# Deep sequencing of the HIV-1 polymerase gene for characterisation of cytotoxic T-lymphocyte epitopes during early and chronic disease stages

**DOI:** 10.1186/s12985-022-01772-8

**Published:** 2022-03-28

**Authors:** Paballo Nkone, Shayne Loubser, Thomas C. Quinn, Andrew D. Redd, Arshad Ismail, Caroline T. Tiemessen, Simnikiwe H. Mayaphi

**Affiliations:** 1 Department of Medical Virology, University of Pretoria, Private Bag X323, Gezina, 0031 South Africa; 2 National Institute for Communicable Diseases and Faculty of Health Sciences, University of the Witwatersrand, Johannesburg, South Africa; 3 Division of Intramural Research, National Institute of Allergy and Infectious Diseases, National Institutes of Health, Bethesda, MD USA; 4 Department of Medicine, Johns Hopkins University, Baltimore, MD USA; 5 National Health Laboratory Service-Tshwane Academic Division (NHLS-TAD), Tshwane, South Africa

**Keywords:** Illumina deep sequencing, Pol CTL epitopes, Early HIV-1 infection, Chronic HIV-1 infection, Minority variants

## Abstract

**Background:**

Despite multiple attempts, there is still no effective HIV-1 vaccine available. The HIV-1 polymerase (*pol*) gene is highly conserved and encodes cytotoxic T-lymphocyte (CTL) epitopes. The aim of the study was to characterise HIV-1 Pol CTL epitopes in mostly sample pairs obtained during early and chronic stages of infection.

**Methods:**

Illumina deep sequencing was performed for all samples while Sanger sequencing was only performed on baseline samples. Codons under immune selection pressure were assessed by computing nonsynonymous to synonymous mutation ratios using MEGA. Minority CTL epitope variants occurring at $\ge$ 5% were detected using low-frequency variant tool in CLC Genomics. Los Alamos HIV database was used for mapping mutations to known HIV-1 CTL epitopes.

**Results:**

Fifty-two participants were enrolled in the study. Their median age was 28 years (interquartile range: 24–32 years) and majority of participants (92.3%) were female. Illumina minority variant analysis identified a significantly higher number of CTL epitopes (n = 65) compared to epitopes (n = 8) identified through Sanger sequencing. Most of the identified epitopes mapped to reverse transcriptase (RT) and integrase (IN) regardless of sequencing method. There was a significantly higher proportion of minority variant epitopes in RT (n = 39, 60.0%) compared to IN (n = 17, 26.2%) and PR (n = 9, 13.8%), *p* = 0.002 and < 0.0001, respectively. However, no significant difference was observed between the proportion of minority variant epitopes in IN versus PR, p = 0.06. Some epitopes were detected in either early or chronic HIV-1 infection whereas others were detected in both stages. Different distribution patterns of minority variant epitopes were observed in sample pairs; with some increasing or decreasing over time, while others remained constant. Some of the identified epitopes have not been previously reported for HIV-1 subtype C. There were also variants that could not be mapped to reported CTL epitopes in the Los Alamos HIV database.

**Conclusion:**

Deep sequencing revealed many Pol CTL epitopes, including some not previously reported for HIV-1 subtype C. The findings of this study support the inclusion of RT and IN epitopes in HIV-1 vaccine candidates as these proteins harbour many CTL epitopes.

**Supplementary Information:**

The online version contains supplementary material available at 10.1186/s12985-022-01772-8.

## Introduction

The HIV/AIDS pandemic has been a global crisis for four decades [1, 2]. At the end of 2020, there were 37.6 million people living with HIV globally, 1.5 million new HIV-1 infections, and 690,000 AIDS-related deaths [3]. South Africa has 7.9 million people living with HIV-1 (PLWH), making it the country with the highest number of infections in the world [4, 5]. This highlights the need to better understand HIV-1 natural immune responses in order to bolster the efforts of developing effective HIV-1 vaccines.

During early HIV-1 infection, effective cytotoxic T-lymphocyte (CTL) immune responses play an important role in the control of viraemia, by contributing to suppression of the viral load (VL) to a set-point [6]. CTL responses target and kill virus-infected cells, via recognition of viral peptide epitopes and releasing cytokines and cytotoxic granules that facilitate cell killing [7–9]. Viral peptide epitopes are presented on the surface of infected cells by the human leukocyte antigen type I (HLA I) trans-membrane proteins encoded by *HLA-A*, *B* and *C* alleles [10–12]. The targeted epitopes undergo immune selection pressure, which leads to the emergence of viral escape mutations [13, 14]. This results in evasion of the immune system, loss of immune control and ultimately progression to AIDS [7, 10]. The majority of epitopes that play a role in the control of viraemia have been extensively studied and reported for HIV-1 Gag [15, 16]. However, some studies show that HIV-1 Pol also harbours important CTL epitopes that contribute to the control of HIV-1 viraemia [17–19], hence *pol* is included in some HIV-1 vaccine candidates [20–25]. There are limited data on the characterisation of HIV-1 Pol CTL epitopes in early and chronic HIV-1 disease stages. The aim of this study was to characterise and assess the evolution of CTL epitopes encoded by HIV-1 *pol* during early and chronic stages of HIV-1 infection, using deep sequencing methods.

## Materials and methods

### Study population

This was a retrospective study that used stored plasma samples that were collected from individuals in the Tshwane district of South Africa. Participants enrolled in this study had a confirmed diagnosis of early or chronic HIV-1 infection and were antiretroviral therapy (ART) naïve. They were identified in a study that screened for early HIV-1 infection in individuals who had a negative rapid test at point-of-care facilities, and diagnosis was confirmed through HIV-1 VL, antibody (enzyme-linked immunoassay), p24 antigen, Western blot and avidity testing [26]. Most of the participants were followed up, and thus samples at two time-points were available (at baseline and follow-up) for analysis.

### Nucleic acid extraction and amplification of HIV-1 *pol*

Total nucleic acids were manually extracted from plasma samples using the QIAamp UltraSens Virus kit (Qiagen, Hilden, Germany). Samples with a VL > 1000 copies/ml were extracted from a plasma input volume of 500 µl, which was adjusted to 1 ml using phosphate-buffered saline (PBS). Samples with a VL ≤ 1000 copies/ml were extracted directly from 1 ml plasma input volume to increase nucleic acid yield. Extraction was performed according to the manufacturer’s instructions except for the first centrifugation step, which was optimized to 800 relative centrifugal force (RCF) instead of the recommended 1200 RCF. This modification facilitated a more efficient dissolution of the pellet. Nucleic acids were eluted in a volume of 60 µl and all the eluates were stored at − 70 °C immediately after extraction.

The complete HIV-1 *pol* gene was amplified in all samples using an in-house nested PCR method, employing the SuperScript™ III One-Step RT-PCR System with Platinum™ *Taq* High Fidelity DNA Polymerase (Invitrogen, Carlsbad, California, USA). Each PCR reaction was performed in a 50 µl reaction, which included 5 µl of extracted RNA / DNA template, 2X reaction mix containing 2.4 mM magnesium sulphate (MgSO_4_), 0.4 mM of each deoxynucleotide triphosphate (dNTPs), 10 mM sense primer, 10 mM anti-sense primer, 5 units (U) of enzyme mix for (first round) or 1U of Platinum Taq polymerase (for second round), and nuclease-free water. The first-round amplicon was used as template for the second-round amplification (www.lifetechnologies.com) (Additional file 1: Table S1). One or two extracted HIV-1-negative controls were included in each experiment to assess potential contamination.

The following sets of primers were used for amplification of the complete *pol* gene: SM-F1 (outer forward) 5′-GCG GCT ACA TTA GAA GAA ATG ATG-3′ (HXB2 1807–1830) and SM-R1 (outer reverse) 5′-GCC AAG TAT TGT AGA GAT CCT ACC T-3′ (HXB2 5462–5488), SM-F3 (inner forward) 5′-AGA TTG TTA AAT GTT TCA ACT GTG G-3′ (HXB2 1952–1976) and SM-R3 (inner reverse) 5′-CTC CTG TAT GCA AAC CCC AAT A-3′ (HXB2 5245–5266). The nested primers amplified a fragment size of 3268 bp.

### Sanger sequencing and sequence analysis

Sanger sequencing was performed on PCR amplicons, but only for baseline samples. Sequencing was performed commercially in six overlapping regions covering the entire *pol* ORF (Inqaba Biotechnical Industries, Tshwane, South Africa) (Additional file 1: Table S1). Sequences were edited in CLC Main Workbench 21 software (Qiagen, Hilden, Germany) and consensus sequences were generated, viewed in BioEdit 7.2.5 (https://bioedit.software.informer.com/download/) and aligned using the online version of the MAFFT program (https://mafft.cbrc.jp/alignment/server/). The HIV-1 HXB2 reference sequence (K03455.1) was used for nucleotide numbering. Phylogenetic analysis was performed on the multiple alignment and the ratio of nonsynonymous to synonymous (dN-dS) mutations within the HIV-1 Pol region was computed using MEGA software (https://www.megasoftware.net/). A dN-dS ratio $\ge$ 5 was used to identify codons under high selection pressure and these sites were subsequently mapped to CTL epitopes documented in the Los Alamos HIV database [27].

### Illumina sequencing and analysis of consensus sequences

Sequencing of PCR amplicons was performed at Inqaba Biotechnical Industries (Pretoria, South Africa), a commercial next generation sequencing (NGS) service provider. Briefly paired-end libraries (2 × 300 bp) were prepared using the NEBNext® Ultra™ II DNA library prep kit for Illumina® (New England Biolabs, USA) and sequencing was performed on an Illumina MiSeq instrument (Illumina, USA).

Some samples (n = 12) that were viewed to have poor quality scores were sent for repeat sequencing to the National Institute for Communicable Diseases (NICD), Sequencing Core Facility, Johannesburg, South Africa. Multiplexed paired-end libraries (2 × 300 bp) were prepared using the Nextera™ XT DNA Library Sample Preparation kit (Illumina Inc., California, United States) according to the manufacturer’s instructions and sequencing was carried out on an Illumina MiSeq instrument (Illumina Inc., California, United States).

Illumina sequencing data was analysed in the PASeq program (https://paseq.org/) using the feature that excludes APOBEC-induced hypermutations, and consensus sequences were generated. The quality of the Illumina reads was assessed in PASeq and CLC Genomics Workbench 21 (Qiagen, Hilden, Germany). PASeq-generated Illumina consensus sequences were viewed in BioEdit 7.2.5 software and aligned to HIV-1 reference sequences obtained from the GenBank database, in the MAFFT program available online. Sanger and Illumina consensus sequences obtained from baseline samples were compared through phylogenetic analysis in MEGA software and were observed to correctly cluster together (Additional file 4: Figure S1).

### Analysis of Illumina sequencing reads for minority variants

Deep sequencing reads were mapped to an HIV-1 subtype C reference (AY162225.1) in CLC Genomics. Minority variants were analysed using a low-frequency variant detection tool in CLC Genomics, setting the cut-off for significance at 1% and the minimum frequency for variants at 5%. Minority variants were assessed in all participants and only nonsynonymous variants were mapped to HIV-1 CTL epitopes in the Los Alamos HIV database, to identify the epitope they fall within. Thus, in this study, minority variant epitopes were defined as epitopes existing at proportions above 5% but less than 20%. The Los Alamos HIV database was also used to predict human leukocyte antigen (HLA) allotypes that may be involved in presenting the identified epitopes to CTLs. Evolution within Pol CTL epitopes was assessed by comparing the proportion of minority variants between baseline and follow-up samples, and in early and chronic HIV-1 disease stages.

### Data analysis

Descriptive statistics was used to present median values and interquartile range (IQR) for age, HIV-1 VL and sequencing depth. Fisher’s exact test was used to assess if there was an association between the distribution of mutations and HIV-1 disease stage for epitopes identified through Sanger sequencing. Two sample t-test was used for comparing the proportions of minority variant epitopes among the different regions of the Pol protein. A p-value of ≤ 0.05 was considered statistically significant. All statistical tests were performed on the STATA 16.0 software package (StataCorp LP, College Station, TX, USA). The statistical analyses of dN–dS ratios were computed on the MEGA programme using the HyPhy test for selection. Due to the small sample size, codons with dN–dS ratios of ≥ 5 were considered for assessment of epitopes under immune selection pressure. GraphPad Prism v.9.1.2 (GraphPad Software, San Diego, California, USA) was used to prepare graphs for the frequently recognised minority variant epitopes among the different regions of the Pol protein, and also for showing the dynamics of these epitopes in sample pairs obtained during early and chronic HIV-1 infection stages.

## Results

### Demographics

This study enrolled 52 participants with HIV-1 infection, 34 of whom had paired plasma samples. Their median age was 28 years (IQR: 24–32 years), and majority were females (92.3%). There were 15 participants (28.9%) with early HIV-1 infection and 37 (71.1%) with chronic HIV-1 infection. The median HIV-1 VL at baseline was 2.8 × ${10}^{4}$ copies/mL (IQR: 8.6 × ${10}^{3}$–9.1 × ${10}^{4}$ copies/mL). The median interval between baseline and follow-up sampling was 4 weeks (IQR: 3–8 weeks) (Table 1).

**Table 1:** Study participants’ demographic data

					Baseline sequencing			Follow-up sequencing			
Sample ID	Sex	Age (years)	HIV Stage	HIV VL	Total reads	HIV reads	Sequencing depth (x)	FU interval (weeks)	Total reads	HIV reads	Sequencing depth (x)
261	M	33	E	8.4 × ${10}^{7}$	415,560	198,157	17,408	No FU			
2504	F	24	E	3.7 × ${10}^{4}$	396,640	288,841	25,452	2	483,620	308,253	27,268
3469	F	20	E	3.3 × ${10}^{4}$	79,948	38,927	3139	9	181,956	104,874	9169
5041	M	23	E	2.2 × ${10}^{7}$	146,352	108,572	10,023	No FU			
6512	F	23	E	1.7 × ${10}^{3}$	155,222	114,138	10,269	2	246,184	181,098	16,380
6727	F	28	E	4.8 × ${10}^{3}$	91,218	60,365	5230	2	202,824	127,955	11,522
6638	F	28	E	1.9 × ${10}^{5}$	167,878	108,348	9913	6	309,802	222,628	20,221
6582	F	24	E	6.2 × ${10}^{3}$	311,276	201,897	17,355	6	857,888	602,248	49,753
9498	F	34	E	5.0 × ${10}^{5}$	145,514	100,637	8871	No FU			
9049	F	20	E	1.6 × ${10}^{4}$	363,168	226,752	19,829	3	1,724,070	1,406,644	65,497
8575	F	27	E	9.3 × ${10}^{4}$	355,040	238,087	21,126	8			
8047	M	31	E	1.2 × ${10}^{6}$	386,426	308,249	28,180	2	559,602	452,725	41,274
7084	F	28	E	3.3 × ${10}^{8}$	199,022	113,257	9843	4	100,980	81,776	7168
6743	F	26	E	2.7 × ${10}^{4}$	87,982	43,575	3928	7	223,468	149,257	78,503
6737	F	24	E	2.2 × ${10}^{3}$	270,528	192,684	16,793	No FU			
639	F	30	C	6.5 × ${10}^{3}$	851,104	651,917	60,785	7	408,144	293,996	25,994
641	F	30	C	7.0 × ${10}^{4}$	1,334,058	969,199	45,128	4	153,448	113,391	10,062
843	F	21	C	2.9 × ${10}^{4}$	328,480	278,706	16,615	5	1,458,950	1,250,697	58,236
921	F	44	C	9.7 × ${10}^{3}$	919,452	599,019	49,637	No FU			
1121	F	27	C	8.0 × ${10}^{4}$	126,868	90,889	8203	2	394,752	283,697	25,273
1213	F	36	C	3.2 × ${10}^{4}$	279,080	228,046	14,505	No FU			
1475	F	32	C	4.4 × ${10}^{4}$	1,165,614	772,938	35,990	9	276,200	186,658	16,450
2340	F	22	C	1.4 × ${10}^{4}$	3,144,374	2,260,428	119,694	No FU			
2678	F	30	C	2.2 × ${10}^{5}$	406,036	291,734	16,510	4	1,300,644	1,060,811	49,394
2696	F	18	C	3.9 × ${10}^{2}$	2,426,068	1,624,274	137,760	No FU			
3253	F	28	C	8.9 × ${10}^{4}$	1,342,710	893,218	41,590	3	2,060,282	1,337,210	115,839
3387	F	37	C	7.9 × ${10}^{4}$	1,769,858	1,107,226	95,026	No FU			
3474	F	21	C	1.6 × ${10}^{4}$	241,256	164,637	14,848	12	289,220	194,533	17,234
9986	F	28	C	9.7 × ${10}^{4}$	187,066	116,633	9830	2	90,100	57,543	4772
3606	F	24	C	1.5 × ${10}^{4}$	558,718	186,290	19,453	No FU			
3869	F	32	C	2.1 × ${10}^{5}$	1,413,006	905,554	42,165	4	1,678,940	1,181,291	98,302
3880	F	30	C	7.5 × ${10}^{3}$	1,569,698	1,138,834	101,974	3	2,339,288	1,600,920	132,141
3910	F	33	C	2.8 × ${10}^{4}$	929,580	715,365	33,309	3	2,858,950	1,977,216	157,648
3912	F	27	C	3.2 × ${10}^{4}$	167,708	120,889	10,826	8	364,208	270,552	23,731
3920	F	20	C	6.6 × ${10}^{4}$	558,718	186,290	16,933	8	741,498	533,342	44,809
3935	F	33	C	2.4 × ${10}^{5}$	1,733,492	1,367,261	63,663	8	3,256,998	1,870,279	130,685
4351	F	35	C	2.6 × ${10}^{3}$	1,091,330	855,414	39,830	3	114,350	71,708	5819
4198	F	19	C	1.7 × ${10}^{3}$	1,523,160	925,214	77,596	No FU			
5054	F	26	C	2.7 × ${10}^{4}$	392,770	294,322	26,607	2	323,594	241,595	21,969
6380	F	25	C	1.1 × ${10}^{4}$	354,870	254,132	23,050	4	229,160	156,127	13,011
6565	F	28	C	5.6 × ${10}^{3}$	1,827,778	1,280,993	76,473	3	121,716	95,006	8145
6671	F	35	C	1.4 × ${10}^{4}$	935,030	676,975	40,433	No FU			
6649	F	32	C	2.1 × ${10}^{4}$	1,163,568	755,545	65,939	2	1,482,096	998,909	83,275
6640	F	37	C	3.0 × ${10}^{3}$	376,388	248,289	21,017	5	1,471,394	1,041,570	93,224
6596	F	31	C	3.8 × ${10}^{3}$	1,318,526	1,082,538	50,406	8	1,346,566	910,739	78,503
6509	M	36	C	1.0 × ${10}^{5}$	655,472	520,480	23,577	No FU			
9915	F	30	C	1.4 × ${10}^{4}$	489,752	372,610	21,830	No FU			
9895	F	31	C	4.8 × ${10}^{3}$	458,108	328,446	18,433	No FU			
9854	F	20	C	1.2 × ${10}^{4}$	393,220	336,159	20,018	No FU			
8828	F	27	C	4.1 × ${10}^{4}$	1,331,066	985,767	45,900	14	94,018	59,609	5106
7959	F	40	C	1.4 × ${10}^{5}$	538,128	453,795	23,053	No FU			
6990	F	26	C	1.7 × ${10}^{4}$	143,560	101,490	9056	6	179,342	136,949	12,630

ID – identity, M = male; F = female; HIV = human immunodeficiency virus; E = early HIV infection; C = chronic HIV infection; VL = viral load; x = times; FU = follow-up

### Amplification of HIV-1 *pol* and phylogenetic analysis

**Fig. 1:**
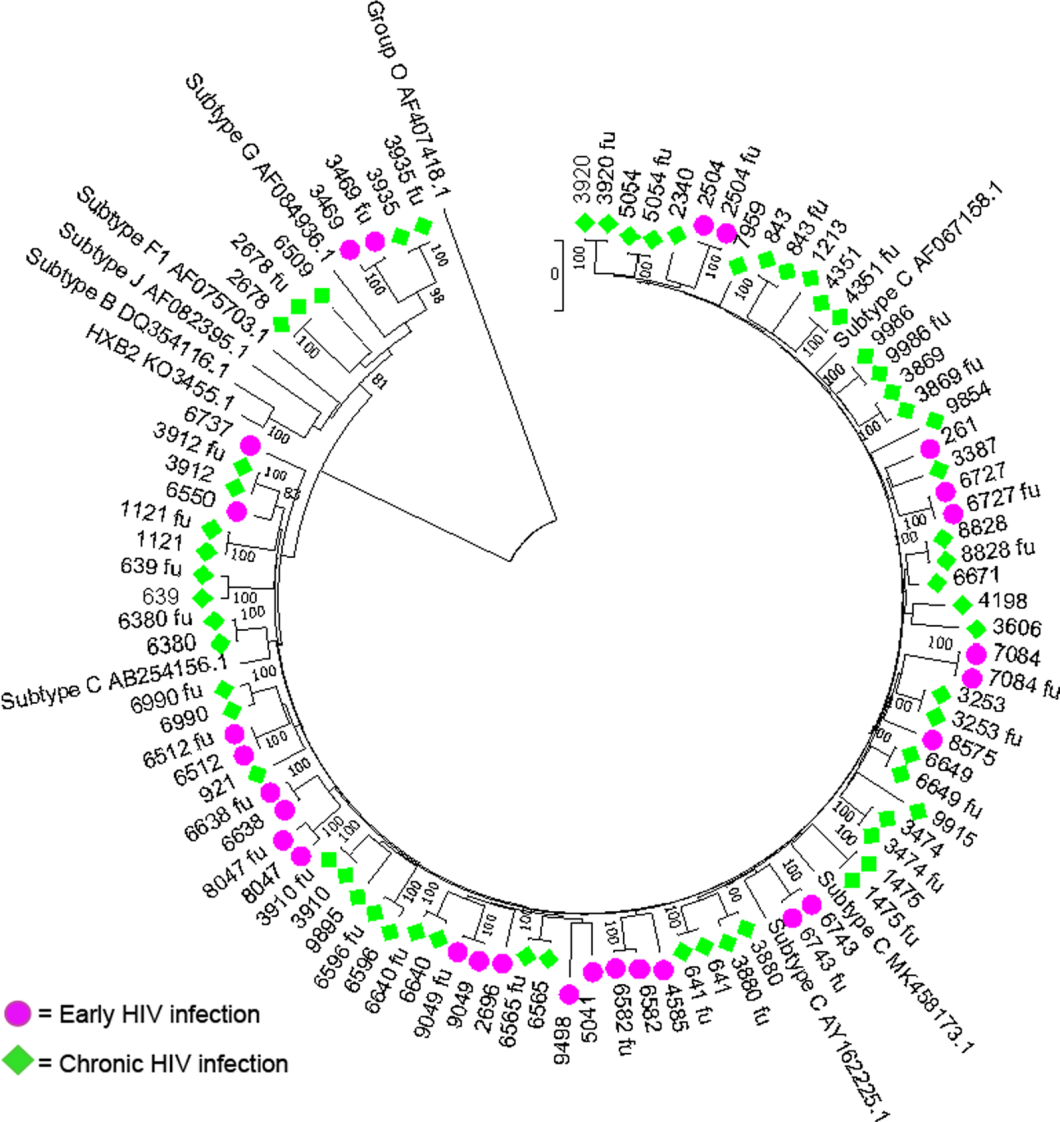
Neighbour-joining phylogenetic tree of all samples including sample pairs. Group O strain of HIV-1 was used for rooting. Only bootstrap values above 70% are shown. Baseline and follow-up (fu) sequences of the same participant correctly clustered

The complete HIV-1 *pol* gene was successfully amplified in all project samples using the in-house nested PCR method. Phylogenetic analysis of Illumina consensus sequences showed that most study sequences clustered with HIV-1 subtype C strains. All baseline and follow-up sequences (OK127761 – OK127810 and OK275500 – OK275537, respectively) for each participant correctly clustered together (Fig. 1), showing accurate amplification and sequencing of viral genes from the same participant.

### Pol CTL epitopes based on Sanger consensus sequences

Eight CTL epitopes with amino acid residues under high selection pressure were identified. The majority of the identified epitopes were located within RT. The distribution of escape mutations was comparable between early and chronic HIV-1 disease stages (Table 2). However, these escape mutations were identified mostly in participants with chronic HIV-1 infection (Additional file 2: Table S2). One or more escape mutations were observed in each epitope. Two other codons were identified to be under high selection pressure but could not be mapped to the reported CTL epitopes in the Los Alamos HIV database (Table 2).

**Table 2:** Nonsynonymous mutations in Pol CTL epitopes assessed from Sanger consensus sequences

				Distribution of mutation by stage of infection		
*pol* protein	Epitope position	Wild type CTL epitope	Escape CTL mutation	Early HIV samples: n = 15 (%)	Chronic HIV samples: n = 34 (%)	*p* value
**PR**	PR (11 – 20)	VTIK**I**GGQLK	**I**15**V**	73,3	59.4	0.345
	PR (30 – 38)	DTVLED**M**NL	**M**36**I/L**	98	79	0.702
**RT**	RT (33 – 41)	AL**V**EICTEM	**V**35**T/K/M**	100	100	N/A
	RT (202 – 210)	**I**EELRQHLL	**I**202**V**	10	17.7	1.000
	RT (269 – 277)	QIY**A**GIKVK	**A**272P/G/S*	80	55	0.345
	Not within epitope RT 329	**I**	**I**329**V/L**	20	5.8	0.160
	RT (375 – 383)	**I**AMESIVIW	**I**375**V**	20	11.8	0.660
**IN**	Not within epitope IN 206	**T**	**T**206**S**	20	15	0.687
	IN (218 – 227)	T**K**IQNFRVYY	**K**219**N/Q**	10	17.6	1.000
	IN (278 – 288)	DDCVA**S**RQDED	**S**283**D/G**	100	95	1.000

^*^Codon had significant dN/dS ratio (*p* value = 0.001). CTL = cytotoxic T-lymphocyte; N/A = not applicable; PR = protease; RT = reverse transcriptase; IN = integrase; Pol = polymerase

### Pol CTL epitopes based on analysis of minority variants

**Fig. 2:**
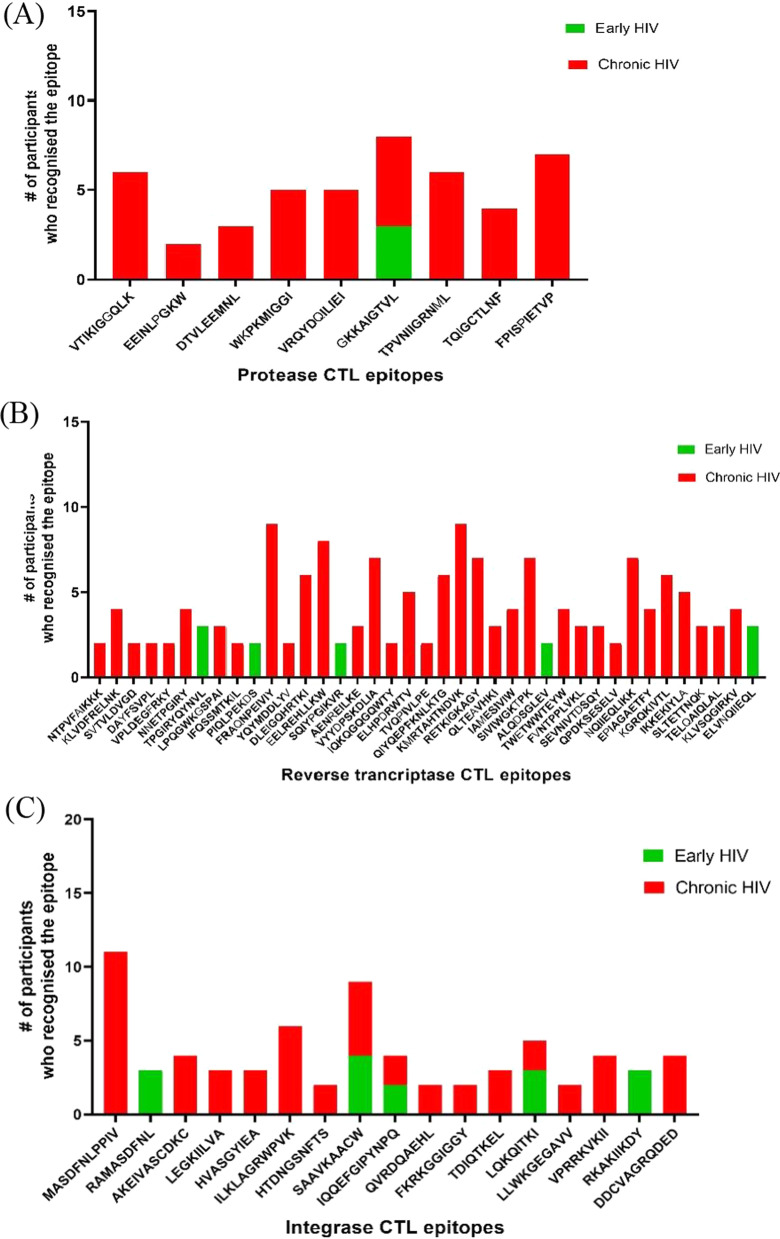
Frequently recognized Pol CTL epitopes in early and chronic HIV infection. These are epitopes that were recognized by more than one participant. **A** Epitopes within the protease (PR) region. **B** Epitopes within the reverse transcriptase (RT) region. **C** Epitopes within the integrase (IN) region. Many epitopes were located within RT and IN as compared to PR, and most were found in chronic HIV infection. CTL = cytotoxic T-lymphocytes

**Fig. 3:**
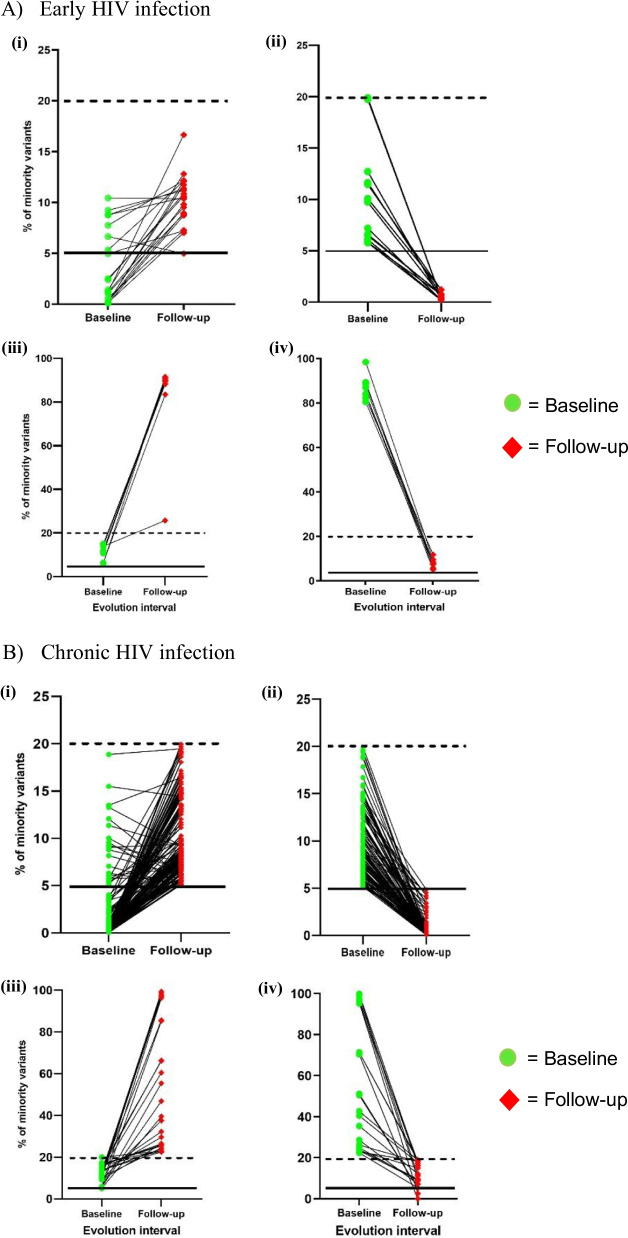
Dynamics of minority variants in sample pairs during **A** early HIV infection and **B** chronic HIV infection. (i) Some variants either increased in proportion between baseline and follow-up or remained at / above the detection limit of 5% (black solid line). (ii) Other variants decreased in proportion over time and were detected below 5% at follow-up. (iii) There were variants that increased in proportion over time to become majority variants and were thus maintained above 20% (black dotted line). (iv) Some minority variants that were detected at follow-up existed as majority variants at baseline. The median interval between baseline and follow-up sampling was 4 weeks (IQR: 3–8 weeks)

The median sequencing depth (for baseline and follow-up) was 23052X (IQR: 13,011–49753X) (Table 1). Minority variant analysis identified 65 frequently targeted Pol CTL epitopes. There was a significantly higher proportion of epitopes in RT (n = 39, 60.0%) compared to IN (n = 17, 26.2%) and PR (n = 9, 13.8%), *p* = 0.002 and < 0.0001, respectively. However, no significant difference was observed between the proportion of minority epitopes in IN versus PR, *p* = 0.06. The majority of these epitopes were identified in participants with chronic HIV-1 infection compared to those with early HIV-1 infection. Some epitopes were only identified in either early or chronic HIV-1 infection, whereas others were identified in both stages of disease (Fig. 2). Different distribution patterns of minority variant epitopes were observed. Some variant epitopes increased or decreased between baseline and follow-up, while others remained constant between these two time-points (Fig. 3, Additional file 3: Table S3). There were minority variants (2 in early and 26 in chronic HIV-1 infection) that could not be mapped to the reported CTL epitopes in the Los Alamos HIV database (Additional file 3: Table S3).

### Pol CTL epitopes and predicted HLA class I presentation

HLA class I allotypes that possibly present the identified epitopes were predicted from the Los Alamos HIV database. Some participants recognised more epitopes than others. This was observed for participants 8047 and 6743 who each recognised seven epitopes during early HIV-1 infection. The most commonly targeted epitope during early HIV-1 infection was SAAVKAACW (IN 123–131), located in IN, and possibly presented by HLA-B*58:01 [27]. The same trend was noticed in chronic HIV-1 infection where the most frequently targeted epitope, MASDFNLPPIV (IN 22–31), was also located in IN and possibly presented by HLA-A*02 [27]. Some of the frequently targeted epitopes identified in this study have never been reported for HIV-1 subtype C. HLA class I allotypes that possibly present the identified epitopes in this study have been reported previously in the South African population [11, 12, 28–32]. Some epitopes were predicted to be recognised by multiple HLA class I allotypes. There were also frequently recognised epitopes that seemed to have similar HLA class I restriction such as ALQDSGLEV (RT 485–494) and RAMASDFNL (IN 20–28) in early HIV-1 infection (Table 3).

**Table 3:**
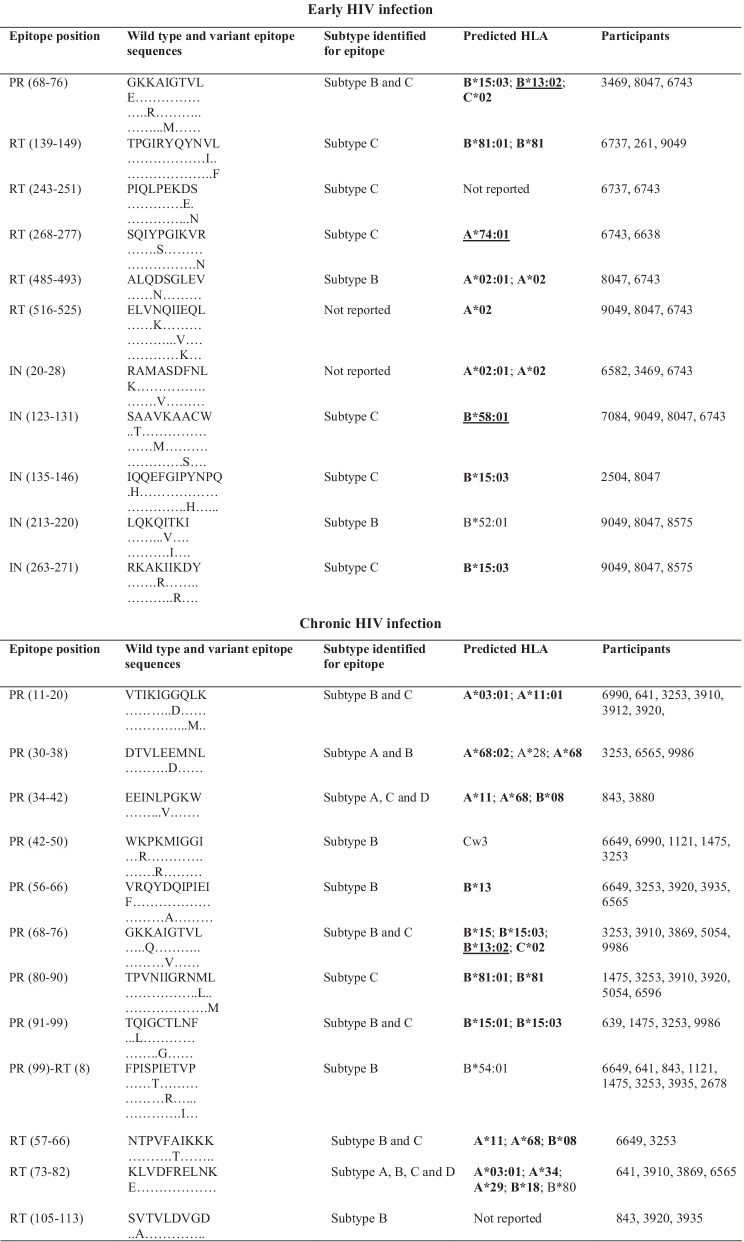

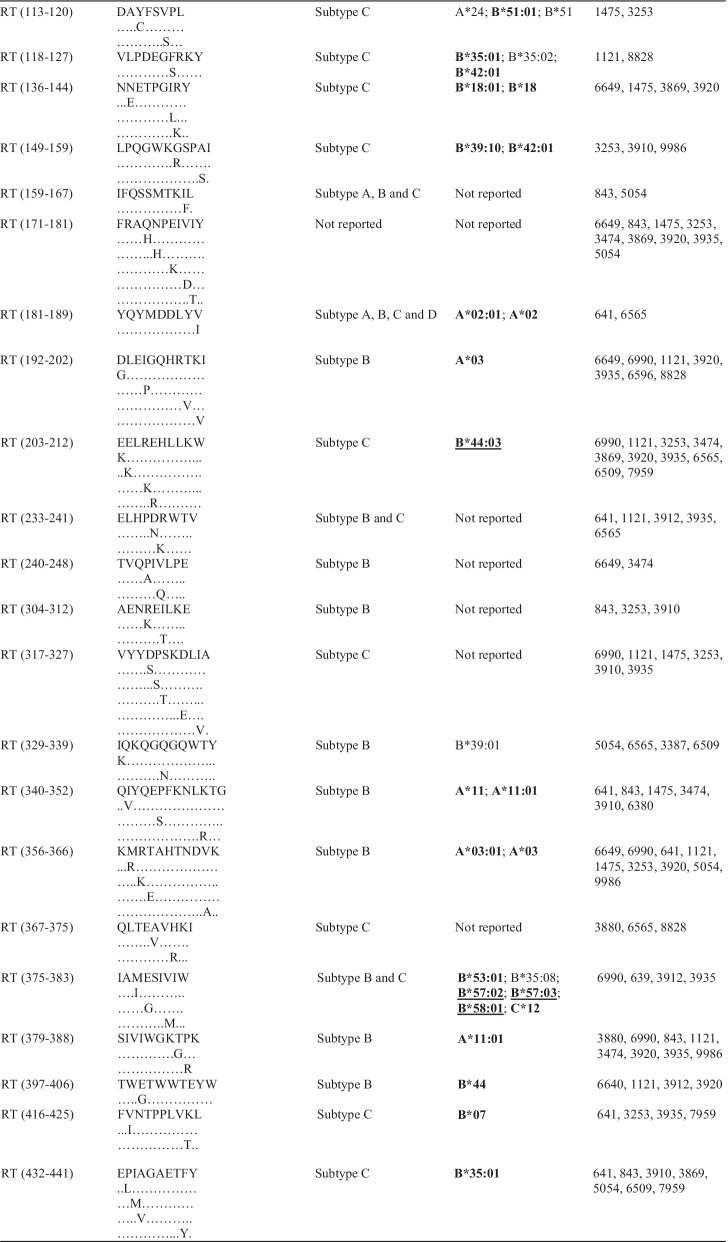

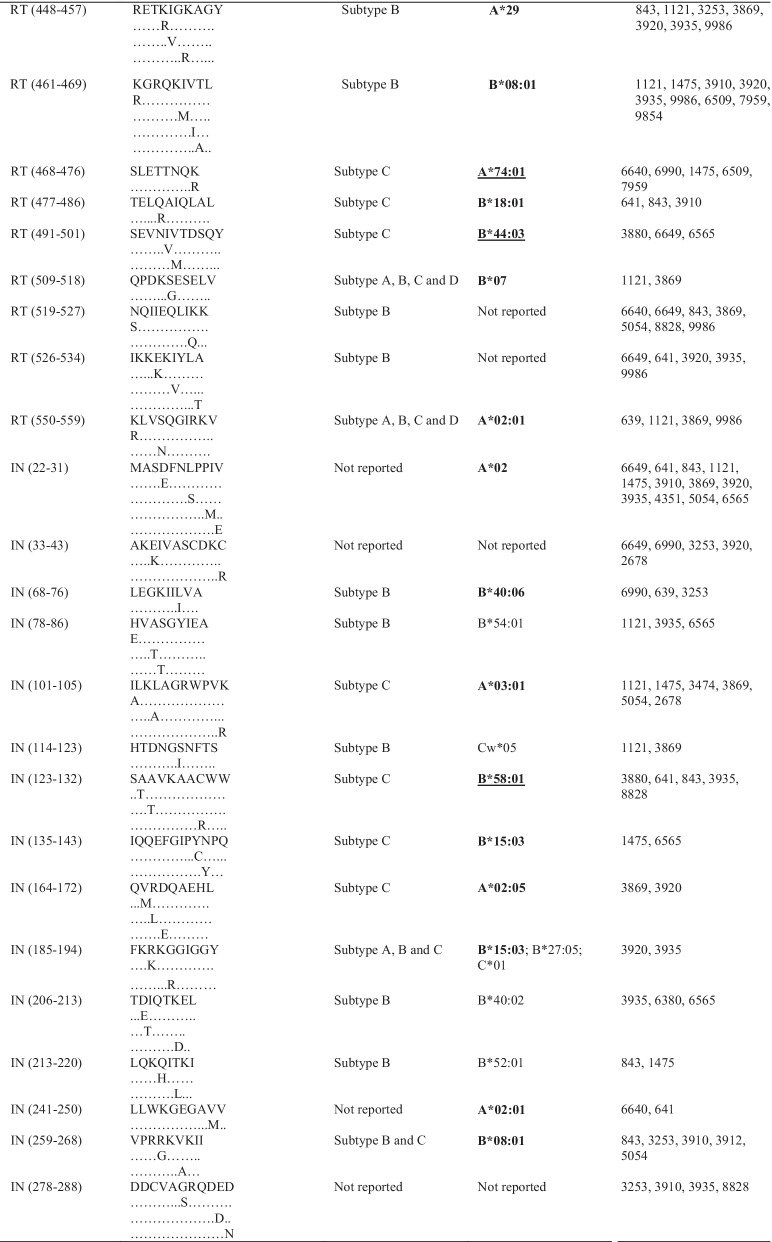
Frequently targeted Pol CTL epitopes and their predicted HLA alleles

HLA alleles in boldface are those that have been identified (reported) in South Africa or southern Africa [11, 28–30]. Alleles underlined are those that were reported to be protective [11, 28, 29, 49, 50]. PR = protease; RT = reverse transcriptase; IN = integrase; HLA = human leukocyte antigen

## Discussion

To our knowledge, this is the first study to assess the evolution of HIV-1 Pol CTL epitopes in samples obtained during early and chronic disease stages in a setting where HIV-1 subtype C is predominant. We identified epitopes within all three regions of Pol, unlike previous studies that have only characterised epitopes within RT and PR [16, 29, 33]. This highlights the advantage of using a PCR method that amplifies the complete *pol* gene. Baseline and follow-up sequences for each participant correctly clustered together, indicating accurate amplification and sequencing of viral genes belonging to the same participant. It was expected that the majority of sequences would cluster with HIV-1 subtype C as this is the most common subtype in the southern African region [34, 35]. A small number of non-subtype C strains was detected, which could have been introduced through migration, and this has also been reported in previous South African studies [36, 37].

Many epitopes were identified through the analysis of minority variants obtained by deep sequencing compared to majority variants obtained by Sanger sequencing. This shows the advantage of using deep sequencing for characterisation of Pol CTL epitopes as this method can detect variants that exist below 20% of the total virus population, which are not readily detected by Sanger sequencing [38, 39]. Some identified minority variant epitopes have never been reported for HIV-1 subtype C and were possibly missed by previous studies that employed Sanger sequencing. The use of Sanger sequencing in most studies has most likely underestimated the presence of CTL epitopes in Pol. Other studies have also shown increased detection of CTL epitopes when using NGS methods [40, 41]. This shows an advantage in the use of NGS for characterising HIV-1 CTL epitopes in future research studies.

In this study, CTL epitopes were identified in all regions of Pol, but mostly in RT and IN. Some of these epitopes such as GKKAIGTVL (PR 68–76) and IAMESIVIW (RT 375–383) (Table 3) have been previously reported to induce CTL responses [42, 43]. These data indicate that all three regions of Pol are immunogenic and may play a role in the control of viraemia and the establishment of a viral set-point during early HIV-1 infection, in agreement with previous studies that assessed CTL responses against the HIV-1 proteome [42–44]. In a study that assessed CTL responses during acute HIV-1 infection, Kim et al.showed that the larger proportion of targeted epitopes were within Pol as compared to other regions of the HIV-1 proteome, and some of these epitopes stimulated strong CTL responses [42]. Ojwach et al.reported that CTL responses towards epitopes in RT and IN during acute and chronic HIV-1 infection induced mutations that decreased viral replicative fitness, indicating that RT and IN harbour crucial CTL epitopes [45]. Fewer epitopes were identified in PR especially during early HIV-1 infection and this could indicate that this protein harbours more subdominant CTL epitopes that are mostly recognised later during the course of infection [46]. Thus, PR might have a limited role in control of viraemia during early HIV-1 infection. CTL epitopes encoded by *pol* have also been reported to play a role in the control of viraemia in long-term non-progressors (LTNPs) and elite controllers (ECs) [47, 48].

HLA class I allotypes play an important role in the control of HIV-1 viraemia [29]. Several HLA class I alleles that encode allotypes predicted to present epitopes identified in this study are known to be present in the South African population, including HLA-B*58:01 and HLA-A*02 that present SAAVKAACW (IN 123–131) and MASDFNLPPIV (IN 22–31), respectively [28, 30, 49]. Past gene association studies showed that alleles within the *HLA-B* group correlate with greater viral control than alleles in the *HLA-A* and *HLA-C* groups [28, 29, 50]. Payne et al.found that HLA-B*58:01 was associated with slower HIV-1 disease progression and was protective against HIV-1 infection [51], which could explain why some participants such as 8047 and 6743 recognised more epitopes, as they also recognised an epitope reported to be presented by HLA-B*58:01. A hierarchy in epitope presentation may be an explanation for recognition of more epitopes during chronic HIV-1 infection. Immunodominant epitopes could be preferentially presented earlier during the course of infection, followed by presentation of subdominant epitopes later [52]. Immunodominant epitopes are often located in variable domains whereas subdominant epitopes usually map to more conserved regions of the HIV-1 proteome [18, 53]. The HIV-1 *pol* gene is more conserved than *gag* and *env* [54] and therefore harbours more subdominant epitopes [18]. Examples of subdominant responses being more effective in controlling viraemia have been shown in past studies [55, 56].

The evolution of Pol CTL epitopes was monitored by assessing minority variants in sample pairs. Minority variant epitopes that increased in proportion from baseline to follow-up may have replaced the wild-type epitopes over time and facilitated immune escape [57]. Fitness cost is the likely explanation for minority variant epitopes that decreased in proportion over time, and these may have been out-competed by new variants [57]. The proportion of some minority variant epitopes remained constant between the two time-points, and these could also represent variants that do not replicate efficiently and are hence maintained at lower levels within the HIV-1 quasispecies [40, 57]. Some variant sites were located outside of reported epitopes (Additional file 3: Table S3), and these may represent new unreported epitopes or could highlight important adjacent sites that may affect epitope processing and presentation [13, 58]. Viral evolution was also assessed by comparing sequences from early and chronic HIV-1 stages. Data from this study showed that evolution within CTL epitopes began quite early following infection, as epitopes with escape mutations were detected in participants who had early HIV-1 infection [59]. Many epitopes with escape mutations were detected in participants in the chronic HIV-1 stage, indicating that viral evolution of HIV-1 results from immune selection pressure that occurs throughout the course of infection and diverse viral populations evolve that constantly evade immune responses [57, 60].

All three expressed proteins (PR, RT and IN) of Pol harbour CTL epitopes and this highlights the importance of including *pol* in the design of a vaccine candidate [2, 20]. Some researchers have included RT and IN in the design of HIV-1 vaccines [22, 23, 53]; this is supported by the findings of our study as the majority of epitopes mapped to these two proteins. Conserved epitopes have been shown to provide cross-clade protection against HIV-1 infection [2, 18]. Some of the conserved Pol epitopes such as IETVEPVKL (RT 5–12) and SVPLDEGFRK (RT 117–126) (Table 3), that were previously observed in a study that looked at using a vaccine candidate with conserved immunogens were also identified in our study [18]. The immune responses directed towards epitopes in conserved regions are usually subdominant but may provide protection against multiple HIV-1 subtypes [17, 53]. Many studies that included Pol epitopes in vaccine candidates showed stimulation of effective CTL responses [18, 56, 61, 62]. Ahmed et al., showed control of HIV-1 replication in vitro through stimulation of Pol CTL responses by a vaccine that contained subdominant epitopes [56].

Limitations of this study include the small sample size and fewer participants with early HIV-1 infection. The duration of infection in participants with chronic HIV-1 infection was not known. HIV-1 VL was not measured in the participants at follow-up, therefore no correlation between VL and the number of minority variant epitopes detected could be made. Functional CTL responses against identified epitopes were not determined and genomic DNA samples were unavailable to conduct HLA class I genotyping. Longitudinal samples were only obtained at two time-points for most of the participants at a median interval of 4 weeks and this might not be sufficient for assessing viral evolution.

## Conclusions

Deep sequencing revealed many HIV-1 Pol CTL epitopes, including some which have never been reported for HIV-1 subtype C. Immune selection pressure on Pol CTL epitopes was observed during early HIV-1 infection highlighting the possibility that these epitopes might contribute to the control of viraemia and the establishment of a VL set-point. Variable patterns of distribution of epitopes in sample pairs reflect the ongoing generation of escape mutants capable of evading the immune responses throughout the course of infection. The findings of this study support the inclusion of RT and IN in potential HIV-1 vaccine candidates, as these regions harbour the majority of CTL epitopes. Variants that could not be mapped to known epitopes in the Los Alamos HIV database might represent new unreported epitopes. Future research, using deep sequencing, is needed for better characterisation of Pol epitopes as this protein contains highly conserved epitopes that have the potential to provide HIV-1 cross-clade protection.

## Supplementary Information

**Additional file 1. Supplementary Table 1:** The in-house complete HIV pol nested PCR conditions and Sanger sequencing primers.

**Additional file 2. Supplementary Table 2:** Pol CTL epitopes identified through Sanger sequencing and comparison by stage of infection

**Additional file 3. Supplementary Table 3:** Minority variant proportions within Pol CTL epitopes by stage of infection.

**Additional file 4. Supplementary Figure 1:** Neighbour-joining phylogenetic analysis of Illumina consensus and Sanger consensus sequences for baseline samples. Sanger and Illumina consensus of the same sample significantly clustered together. Majority of the sequences clustered with HIV-1 subtype C references. HIV group O was used for rooting the tree and a bootstrap value of 1000 was used for analysis. S = Sanger sequencing; D = Deep sequencing.

## Data Availability

All data generated or analysed during this study are included in this manuscript and its supplementary information files.

## Abbreviations

HIV-1: Human immunodeficiency virus type 1
PLWH: People living with HIV
CTL: Cytotoxic T-lymphocyte
VL: Viral load
HLA I: Human leukocyte antigen type 1
ART: Antiretroviral therapy
RCF: Relative centrifugal force
RT-PCR: Reverse transcriptase polymerase chain reaction
RNA: Ribonucleic acid
DNA: Deoxyribonucleic acid
dNTPs: Deoxynucleotide triphosphate
ORF: Open reading frame
NGS: Next generation sequencing
IQR: Interquartile range
Gag: Group-specific antigen
Pol: Polymerase
Env: Envelope
PR: Protease
RT: Reverse transcriptase
IN: Integrase
LTNP: Long-term non-progressors
EC: Elite controllers
