# Correction to: Deep sequencing of the HIV-1 polymerase gene for characterisation of cytotoxic T-lymphocyte epitopes during early and chronic disease stages

**DOI:** 10.1186/s12985-022-01803-4

**Published:** 2022-05-05

**Authors:** Paballo Nkone, Shayne Loubser, Thomas C. Quinn, Andrew D. Redd, Arshad Ismail, Caroline T. Tiemessen, Simnikiwe H. Mayaphi

**Affiliations:** 1 Department of Medical Virology, University of Pretoria, Private Bag X323, Gezina, 0031 South Africa; 2 National Institute for Communicable Diseases and Faculty of Health Sciences, University of the Witwatersrand, Johannesburg, South Africa; 3 Division of Intramural Research, National Institute of Allergy and Infectious Diseases, National Institutes of Health, Bethesda, MD USA; 4 Department of Medicine, Johns Hopkins University, Baltimore, MD USA; 5 National Health Laboratory Service-Tshwane Academic Division (NHLS-TAD), Tshwane, South Africa

## Correction to: Virology Journal (2022) 19:56 10.1186/s12985-022-01772-8

Following publication of the original article [1], the authors informed us that many epitopes from Table 3 have gross alignment issues, which were probably caused by formatting of this table before publication. The correct table is given below.

**Table 3:**
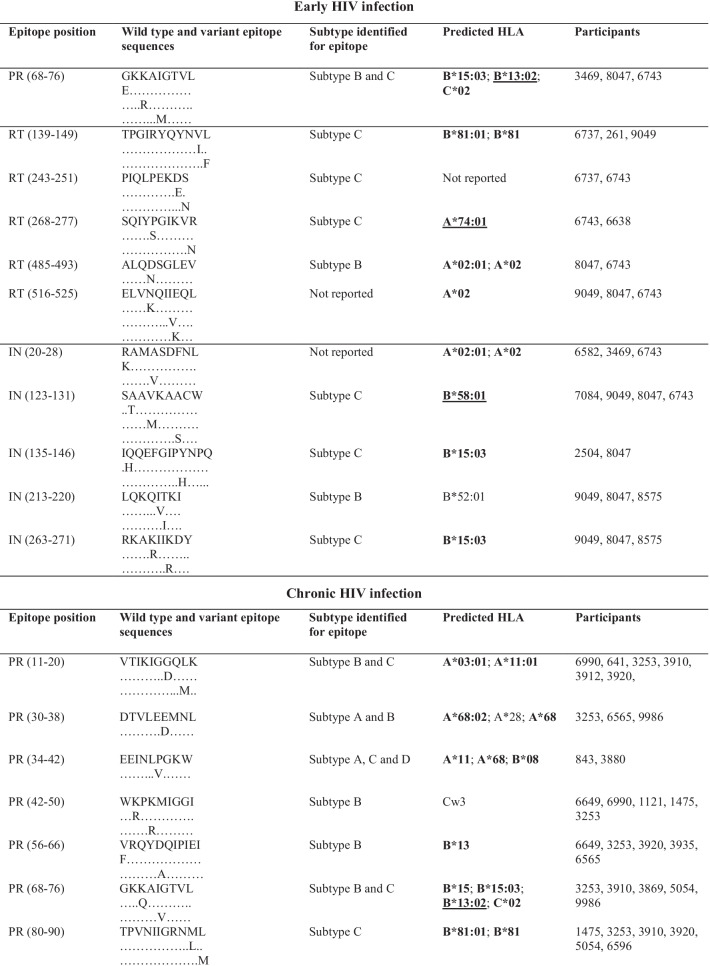

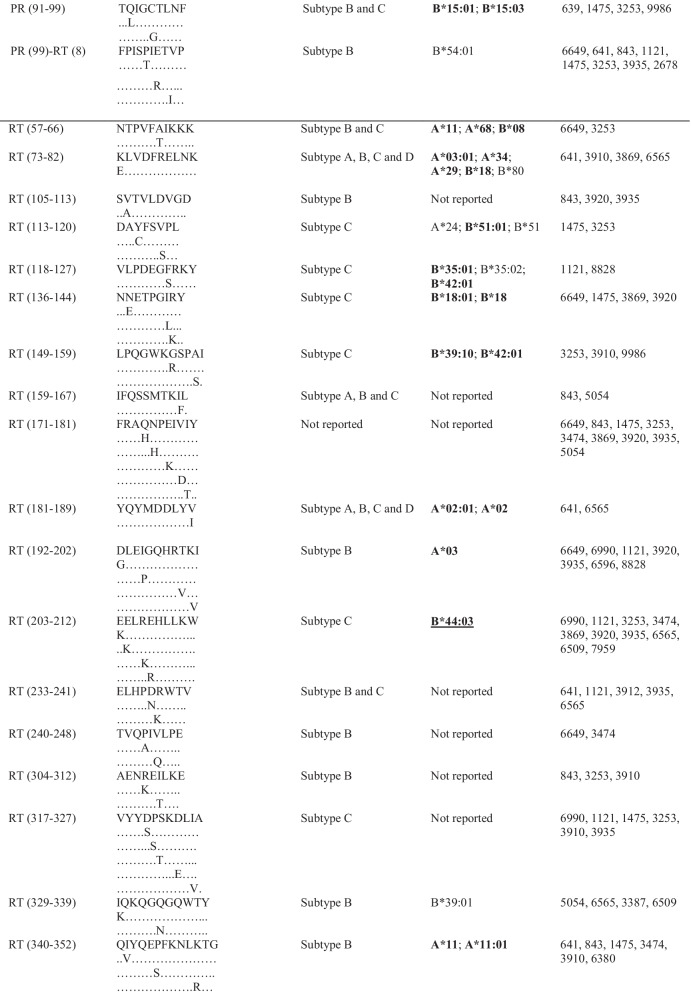

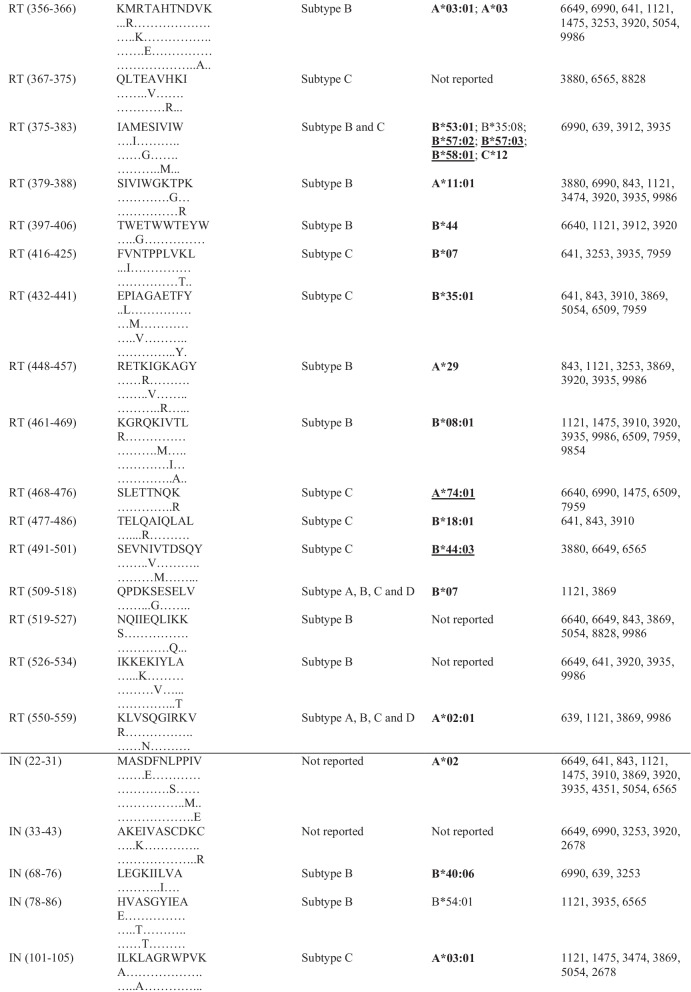

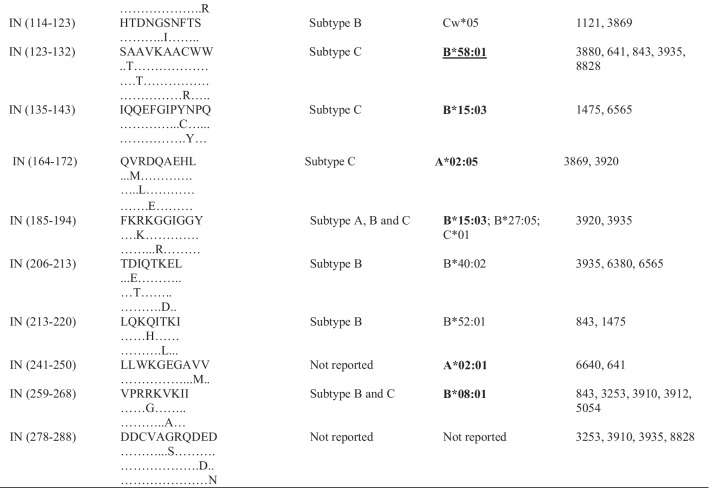
Frequently targeted Pol CTL epitopes and their predicted HLA alleles

HLA alleles in boldface are those that have been identified (reported) in South Africa or southern Africa [11,28–30]. Alleles underlined are those that were reported to be protective [11,28–29,49,50]. PR = protease; RT = reverse transcriptase; IN = integrase; HLA = human leukocyte antigen

The original article has been corrected.
