# Supplementary material for: Deep sequencing of the HIV-1 polymerase gene for characterisation of cytotoxic T-lymphocyte epitopes during early and chronic disease stages
Source: Virol J. 2022 Mar 28;19:56. doi: 10.1186/s12985-022-01772-8 (PMC8959563; doi:10.1186/s12985-022-01772-8)
Supplement: Supplementary file 1 — Additional file 1. Supplementary Table 1: The in-house complete HIV pol nested PCR conditions and Sanger sequencing primers. [file 12985_2022_1772_MOESM1_ESM.docx]

Supplementary Table 1: The in-house complete HIV *pol* nested PCR conditions and Sanger sequencing primers

| **First round** | | **Second round** | |
| --- | --- | --- | --- |
| RT = 55$℃$ for 30 min | |  | |
| Pre-denaturation = 94$℃$ for 2 min | | Pre-denaturation = 94$℃$ for 2 min | |
| 35 cycles | Denaturation = 94$℃$ for 15 sec | 30 cycles | Denaturation = 94$℃$ for 15 sec |
|  | Annealing = 50$℃$ for 30 sec |  | Annealing = 50$℃$ for 30 sec |
|  | Extension = 68$℃$ for 4 min |  | Extension = 68$℃$ for 3.5 min |
| Final extension = 68$℃$ for 10 min | | Final extension = 68$℃$ for 10 min | |
| Infinite hold = 4$℃$ | | | |

RT = reverse transcription; min = minutes; sec = seconds; $℃$ = degree Celsius

Sanger sequencing primers

SM-F3 Alt (forward) 5’-GTT AAG TGT TTC AAC TGT G-3’ (SM-F3 shorter version) and SM-R3 (reverse) were used as outer sequencing primers together with the following inner primers: SM-Int-R1 (reverse) 5’-CTR CAR TCY ACY TGB CCA TG-3’ (4380-99), SM-Int-R2 (reverse) 5’-CCA BTC AGG WAT CCA G-3’ (3776-91), SM-Int-R3 (reverse) 5’-TGD AGY TCR TAY CCC ATC CA-3’ (3234-53). An alternative primer SM-Int-R2 Alt (reverse) 5’-CCA NTC RGG DAY CCA G-3’ was used in few samples that could not be sequenced by SM-Int-R2 due to a mismatch. SM-DF3 and SM-DR3 or SM-F2 and SM-R2 primer pair were used as outer sequencing primers for few samples that were successfully amplified with these primers, together with the above mentioned inner sequencing primers. Sequencing was repeated on the same or newly amplified template for samples that initially had failed sequencing.
