# Supplementary material for: Deep sequencing of the HIV-1 polymerase gene for characterisation of cytotoxic T-lymphocyte epitopes during early and chronic disease stages
Source: Virol J. 2022 Mar 28;19:56. doi: 10.1186/s12985-022-01772-8 (PMC8959563; doi:10.1186/s12985-022-01772-8)
Supplement: Supplementary file 2 — Additional file 2. Supplementary Table 2: Pol CTL epitopes identified through Sanger sequencing and comparison by stage of infection [file 12985_2022_1772_MOESM2_ESM.docx]

**Supplementary Table 2**: Pol CTL epitopes identified through Sanger sequencing and comparison by stage of infection

| **HIV *pol* position** | **Wild type CTL epitope** | **Escape CTL mutation** | | | | | |
| --- | --- | --- | --- | --- | --- | --- | --- |
|  |  | **Early HIV samples: n=15 (%)** | | Participants | **Chronic HIV samples: n=34 (%)** | | Participants |
| PR (11 – 20) | VTIK**I**GGQLK | **I**15**V** | 73.3 | 8047, 6638, 6512, 6743, 7084, 2504, 3469, 261, 9498, 8575, 5041 | **I**15**V** | 59,4 | 639, 843, 1121, 1475, 2678, 3253, 3474, 9986, 3880, 3920, 5054, 6565, 6649, 6640, 4198, 7959, 9915, 9895, 6671, 9854, 3606 |
| PR (30 – 38) | DTVLED**M**NL | **M**36**I/L** | 98 | 8047, 9049, 6638, 6512, 6743, 6582, 6727, 7084, 2504, 3469, 261, 9498, 8575, 6737, 5041 | **M**36**I/L** | 79 | 639, 843, 1121, 1475, 2678, 3253, 3474, 9986, 3880, 3910, 3912, 3920, 3935, 4351, 5054, 6649, 6640, 4198, 6990, 2696, 9915, 9895, 6671, 9854, 3606, 1213, 3387 |
| RT (33 – 41) | AL**V**EICTEM | **V**35**T/K/M** | 100 | All samples | **V**35**T/K/M** | 100 | All samples |
| RT (202 – 210) | **I**EELRQHLL | **I**202**V** | 10 | 6512, 6737 | **I**202**V** | 17,7 | 1475, 6380, 6649, 6509, 1213 |
| RT (269 – 277) | QIY**A**GIKVK | **A**272**P/G** | 80 | 8047, 6512, 6743, 6582, 6727, 7084, 2504, 3469, 261, 9498, 8575, 6737, 5041 | **A**272**P/G/S** | 55 | 843, 1121, 3253, 3474, 9986, 3880, 3910, 3920, 4351, 6380, 6649, 6990, 4198, 7959, 2696, 9915, 9895, 6671, 3387, 921 |
| Not within epitope RT 329 | **I** | **I**329**V/L** | 20 | 9049, 6512, 7084 | **I**329**V/L** | 5,8 | 3912, 1213 |
| RT (375 – 383) | **I**AMESIVIW | **I**375**V** | 20 | 3469, 6737, 5041 | **I**375**V** | 11,8 | 3474, 3935, 4351, 8828, 6509 |
| Not within epitope IN 206 | **T** | **T**206**S** | 20 | 6638, 2504, 3469 | **T**206**S** | 15 | 3910, 3920, 3935, 6671, 1213 |
| IN (218 – 227) | T**K**IQNFRVYY | **K**219**N/Q** | 10 | 6638, 6582 | **K**219**N/Q** | 17,6 | 843, 1475, 3910, 6640, 9915, 9895, 1213 |
| IN (278 – 288) | DDCVA**S**RQDED | **S**283**D/G** | 100 | All samples | **S**283**D/G** | 95 | 639, 843, 1121, 1475, 2678, 3253, 3474, 9986, 3880, 3912, 3696, 3920, 3935, 4351, 5054, 6649, 6640, 4198, 6990, 2696, 9915, 9895, 6671, 9854, 3606, 1213, 3387, 6509, 6596, 6380, 6565, |

PR = protease; RT = reverse transcriptase; IN = integrase; pol = polymerase; CTL = cytotoxic T-lymphocytes.
