# Supplementary material for: Deep sequencing of the HIV-1 polymerase gene for characterisation of cytotoxic T-lymphocyte epitopes during early and chronic disease stages
Source: Virol J. 2022 Mar 28;19:56. doi: 10.1186/s12985-022-01772-8 (PMC8959563; doi:10.1186/s12985-022-01772-8)
Supplement: Supplementary file 3 — Additional file 3. Supplementary Table 3: Minority variant proportions within Pol CTL epitopes by stage of infection. [file 12985_2022_1772_MOESM3_ESM.docx]

**Supplementary Table 3**: Minority variant proportions within Pol CTL epitopes by stage of infection

| **Early HIV Infection** | | | | | | | | |
| --- | --- | --- | --- | --- | --- | --- | --- | --- |
|  |  |  |  | | % of minority variant | | |  |
| **SampleID** | ***pol* location** | **Wild type CTL Epitope** | **Amino acid variant** | | **Baseline*** | | **Follow-up*** | **Subtype identified for** |
| 6582 | IN (20-28) | RAMASDFNL | **A**23**V** | | 12.71 | | 0.26 | Subtype B |
| 6638 | PR (3-11) | ITLWQRPLV | **T**4**A** | | 1.2 | | 16.66 | Subtype A, B and C |
|  | RT (159-167) | IFQSSMTKI | **K**166**N** | | 0.19 | | 12.09 | Subtype A, B and C |
|  | RT (268-277) | SQIYPGIKVR | **R**277**N** | | 4.96 | | 7.23 | Subtype C |
|  | IN (9-19) | QEEHEKYHSNW | **E**10**V** | | 0.23 | | 11.75 | Subtype C |
|  | IN (52-60) | GQVDCSPGI | **I**60**T** | | 1.42 | | 12.82 | Not reported |
|  | IN (259-268) | VVPRRKAKII | **V**259**E** | | 2.42 | | 10.86 | Subtype B and C |
| 7084 | IN (123-131) | SAAVKAACW | **V**126**M** | | 9.74 | | 0.61 | Subtype A, B, C and D |
|  | IN (123-131) | SAAVKAACW | **V**126**M** | | 10.09 | | 1.22 | Subtype A, B, C and D |
| 6737 | PR (42-50) | TPGIRYQYNVL | **V**49**I** | | 6.66 | | N/D | Subtype C |
|  | RT (243-251) | PIQLPEKDS | **S**251**N** | | 14.92 | | N/D | Subtype B and C |
| 2504 | RT (128-135) | TAFTIPSI | **I**132**V** | | 0.18 | | 7.02 | Subtype C |
|  | RT (550-559) | KLVSQGIRKVL | **K**557**R** | | 0.1 | | 8.72 | Subtype B and C |
|  | IN (135-146) | IQQEFGIPYNPQ | **Y**143**H** | | 1.11 | | 8.89 | Subtype C |
|  | IN (197-204) | GERIIDII | **R**199**G** | | 0.63 | | 7.29 | Subtype B |
| 9049 | PR (11-20) | TPVNIIGRNML | **M**19**I** | | 10.45 | | 10.38 | Subtype A and C |
|  | PR (42-50) | TPGIRYQYNVL | **V**49**I** | | 19.9 | | 0.43 | Subtype C |
|  | PR (68-76) | ILKEPVHGV | **V**73**A** | | 0.29 | | 8.96 | Subtype B and D |
|  | RT (461-469) | RGRQKVVSL | **V**466**I** | | 19.74 | | 0.41 | Subtype B |
|  | RT (516-525) | ELVNQIIEQL | **N**519**K** | | 6.48 | | 0.81 | Subtype B and C |
|  | RT (516-525) | ELVNQIIEQL | **I**522**V** | | 6.1 | | 0.21 | Subtype B and C |
|  | IN (62-71) | QLDCTHLEGK | **T**66**I** | | 5.79 | | 0.2 | Subtype C |
|  | IN (123-131) | SAAVKAACW | **A**129**S** | | 82.33 | | 11.79 | Subtype A, B, C and D |
|  | IN (213-220) | LQKQITKI | **I**217**V** | | 6.59 | | 0.78 | Subtype B |
|  | IN (218-227) | TKIQNFRVYYR | **F**223**L** | | 5.81 | | 0.75 | Subtype C |
|  | IN (263-271) | RKAKIIKDY | **K**269**R** | | 7.19 | | 0.2 | Subtype C |
| 261 | PR (42-50) | TPGIRYQYNVL | **L**50**N** | | 5.99 | | N/D | Subtype C |
|  | IN (185-194) | FKRKGGIGGY | **K**188**R** | | 6.44 | | N/D | Subtype B and C |
| 8047 | PR (57-66) | RQYDQILIEI | **Q**61**H** | | 11.68 | | 89.91 | Subtype B |
|  | PR (57-66) | RQYDQILIEI | **L**63**P** | | 98.46 | | 9.61 | Subtype B |
|  | PR (57-66) | RQYDQILIEI | **L**63**P** | | 11.64 | | 0.71 | Subtype B |
|  | PR (68-76) | GKKAIGTVL | **I**72**M** | | 11.46 | | 1.19 | Subtype B and C |
|  | RT (173-183) | KQNPEIVIYQY | **K**173**T** | | 11.87 | | 88.34 | Subtype B and C |
|  | RT (448-457) | RETKIGKAGY | **I**452**L** | | 11.69 | | 89.36 | Subtype B |
|  | RT (448-457) | RETKIGKAGY | **I**452**L** | | 88.68 | | 8.35 | Subtype B |
|  | RT (485-493) | ALQDSGLEV | **D**488**N** | | 11.82 | | 90.1 | Subtype A, B and D |
|  | RT (516-525) | ELVNQIIEQL | **N**519**K** | | 97.4 | | 8.88 | Subtype B and C |
|  | IN (28-36) | LPPIVAKEI | **I**31**V** | | 87.02 | | 8.33 | Not reported |
|  | IN (123-131) | SAAVKAACW | **A**124**T** | | 11.19 | | 90.65 | Subtype A, B, C and D |
|  | IN (123-131) | SAAVKAACW | **A**129**S** | | 10.77 | | 89.7 | Subtype A, B, C and D |
|  | IN (135-146) | IQQEFGIPYNPQ | **Q**136**H** | | 11.2 | | 91.25 | Subtype C |
|  | IN (213-220) | LQKQITKI | **T**218**I** | | 13.94 | | 88.44 | Subtype B |
|  | IN (232-241) | DPIWKGPAKL | **P**233**M** | | 14.97 | | 88.2 | Subtype A, B and C |
|  | IN (267-271) | RKAKIIKDY | **K**269**R** | | 84.12 | | 7.74 | Subtype B and C |
| 8575 | IN (197) | Not within epitope | **L**197**F** | | 5.17 | | N/D |  |
|  | IN (213-220) | LQKQITKI | **I**217**V** | | 6.77 | | N/D | Subtype B |
|  | IN (267-271) | RKAKIIKDY | **K**270**R** | | 6.48 | | N/D | Subtype B and C |
| 6743 | PR (68-76) | GKKAIGTVL | **G**68**E** | | 13.89 | | 25.7 | Subtype B and C |
|  | RT (33-41) | ALVEICTEM | **E**40**K** | | 6.66 | | 4.98 | Subtype B |
|  | RT (192-202) | DLEIGQHRTKI | **K**201**R** | | 84.29 | | 7.41 | Subtype A and B |
|  | RT (206-214) | RAHLLKWGL | **L**214**F** | | 6.34 | | 83.43 | Subtype C |
|  | RT (243-251) | PIQLPEKDS | **D**250**E** | | 6.02 | | 91.53 | Subtype B and C |
|  | RT (268-277) | SQIYPGIKVR | **P**272**S** | | 0.25 | | 9.82 | Subtype A, B and C |
|  | RT (271-279) | YPIGKVRQL | **Q**278**H** | | 2.53 | | 10.62 | Subtype C |
|  | RT (271-279) | YPIGKVRQL | **L**279**F** | | 1.23 | | 9.54 | Subtype C |
|  | RT (317-327) | VYYDPSKDLIA | **D**324**E** | | 80.55 | | 7.72 | Subtype C |
|  | RT 335 | Not within epitope | **M**335**I** | | 8.77 | | 11.39 |  |
|  | RT (357-366) | RRTTHTNDV | **D**365**N** | | 8.79 | | 10.5 | Not reported |
|  | RT (485-493) | ALQDSGLEV | **D**488**N** | | 5.34 | | 12.11 | Subtype A, B and D |
|  | RT (516-525) | ELVNQIIEQL | **E**523**K** | | 7.77 | | 12.17 | Subtype B and C |
|  | IN (20-28) | RAMASDFNL | **R**20**K** | | 9.24 | | 11.19 | Subtype B |
|  | IN (123-131) | SAAVKAACW | **A**124**T** | | 89.4 | | 5.79 | Subtype A, B, C and D |
|  | IN (218-227) | TKIQNFRVYYR | **F**221**L** | | 84.12 | | 5.05 | Subtype B and C |
| **Chronic HIV Infection** | | | | | | | | |
|  |  |  | |  | | % of minority variant | |  |
| **SampleID** | ***pol* location** | **Wild type CTL Epitope** | | **Amino acid variant** | | **Baseline*** | **Follow-up*** | **Subtype identified for** |
| 6990 | PR (11-20) | VTIKIGGQLK | | **G**17**D** | | 17,12 | 23,18 | Subtype B and C |
|  | PR (42-50) | WKPKMIGGI | | **K**43**R** | | 3,66 | 18,61 | Subtype B |
|  | PR (42-50) | WKPKMIGGI | | **K**45**R** | | 7,34 | 0,44 | Subtype B |
|  | PR 53 | Not within epitope | | **A**53**D** | | 17,85 | 0,39 |  |
|  | RT (192-202) | DLEIGQHRTKI | | **I**202**V** | | 9,01 | 0,33 | Subtype A and B |
|  | RT (203-212) | EELREHLLKW | | **E**203**V** | | 11,72 | 0,58 | Subtype C |
|  | RT (317-327) | VYYDPSKDLIA | | **D**324**E** | | 5,07 | 1,19 | Subtype C |
|  | RT (356-366) | KMRTAHTNDVK | | **V**367**A** | | 10,91 | 97,02 | Subtype B |
|  | RT (375-383) | IAMESIVIW | | **M**377**I** | | 8,03 | 1,03 | Subtype B and C |
|  | RT (379-388) | SIVIWGKTPK | | **T**386**G** | | 5,56 | 2,89 | Subtype B |
|  | RT (379-388) | SIVIWGKTPK | | **K**388**R** | | 11,88 | 0,3 | Subtype B |
|  | RT (468-476) | SLTETTNQK | | **K**476**R** | | 15,77 | 1,02 | Subtype C |
|  | RT (485-494) | ALQDSGSEVN | | **Q**487**R** | | 9,25 | 0,81 | Subtype A, B and D |
|  | RT 496 | Not within epitope | | **G**496**Q** | | 13,48 | 0,44 |  |
|  | IN (33-43) | AKEIVASCDKC | | **E**35**K** | | 5,6 | 0,12 | Subtype B |
|  | IN (68-76) | LEGKIILVA | | **L**74**I** | | 10,16 | 0,18 | Not reported |
| 1213 | RT (268-277) | SQIYPGIKVR | | **P**272**S** | | 6.13 | N/D | Subtype C |
|  | RT (271-279) | YPIGKVRQL | | **Q**278**N** | | 6.20 | N/D | Subtype C |
| 3880 | PR (34-42) | EEINLPGKW | | **P**39**V** | | 8,09 | 0,61 | Subtype B and C |
|  | RT (367-375) | QLTEAVHKI | | **H**373**R** | | 0,12 | 12,02 | Subtype A, B and C |
|  | RT (379-388) | SIVIWGKTPK | | **T**386**G** | | 7,59 | 0,79 | Subtype B |
|  | RT (491-501) | SEVNIVTDSQY | | **I**495**V** | | 7,87 | 0,27 | Subtype C |
|  | IN (9-19) | QEEHEKYHSNW | | **E**13**D** | | 7,53 | 0,51 | Subtype B and C |
|  | IN (123-131) | SAAVKAACWW | | **C**129**R** | | 0,15 | 6,31 | Subtype A, B, C and D |
| 2340 | RT (499-506) | SQYALGII | | **S**499**P** | | 11.23 | N/D | Subtype C |
|  | IN (78-86) | HVASGYIEA | | **S**81**T** | | 5.05 | N/D | Not reported |
| 6649 | PR (42-50) | WKPKMIGGI | | **K**45**R** | | 13,8 | 53,42 | Subtype B |
|  | PR (56-66) | VRQYDQIPIEI | | **D**60**A** | | 1,29 | 15,34 | Subtype B |
|  | PR (99)-RT (9) | FPISPIETVP | | **E**6**R** | | 0,65 | 14,22 | Subtype B |
|  | RT (57-66) | NTPVFAIKKK | | **A**62**T** | | 0,79 | 8,99 | Subtype C |
|  | RT (136-144) | NNETPGIRY | | **R**143**K** | | 0,25 | 12,77 | Subtype C and D |
|  | RT (171-181) | FRAQNPEIVIY | | **N**175**H** | | 0,23 | 15 | Subtype A and B |
|  | RT (192-202) | DLEIGQHRTKI | | **D**192**G** | | 0,19 | 9,29 | Subtype A and B |
|  | RT (240-248) | TVQPIVLPE | | **P**243**A** | | 13,6 | 23,97 | Not reported |
|  | RT (240-248) | TVQPIVLPE | | **V**245**Q** | | 13,71 | 23,98 | Not reported |
|  | RT 250 | Not within epitope | | **A**250**D** | | 14,23 | 0,74 |  |
|  | RT (356-366) | KMRTAHTNDVK | | **V**365**A** | | 0,18 | 6,53 | Subtype B |
|  | RT (390-398) | RLPIQKETW | | **T**397**A** | | 0,31 | 13,18 | Subtype B |
|  | RT (397-406) | TWETWWTEYW | | **E**399**G** | | 1,31 | 13,56 | Subtype B |
|  | RT (397-406) | TWETWWTEYW | | **E**399**G** | | 13,37 | 0,57 | Subtype B |
|  | RT (491-501) | SEVNIVTDSQY | | **V**496**M** | | 0,98 | 8,47 | Subtype C |
|  | RT (519-527) | NQIIEQLIKK | | **K**527**Q** | | 13,26 | 0,69 | Subtype B |
|  | RT (526-534) | IKKEKIYLA | | **A**534**T** | | 0,56 | 7,28 | Subtype B |
|  | IN (22-31) | MASDFNLPPIV | | **V**31**E** | | 13,3 | 0,42 | Not reported |
|  | IN (33-43) | AKEIVASCDKC | | **C**43**R** | | 0,31 | 12,9 | Subtype B |
|  | IN 47 | Not within epitope | | **I**47**M** | | 13,28 | 9,22 |  |
|  | IN (173-181) | KTAVQMAVF | | **M**178**T** | | 2,15 | 10,27 | Subtype C |
| 2696 | IN (37-45) | VASCDKCQL | | **C**40**R** | | 7.94 | N/D | Subtype C |
|  | IN (278-288) | DDCVAGRQDED | | **Q**285**R** | | 6.64 | N/D | Not reported |
| 6640 | PR 29 | Not within epitope | | **K**29**G** | | 19,4 | 0,39 |  |
|  | RT 250 | Not within epitope | | **A**250**D** | | 50,47 | 5,32 |  |
|  | RT (468-476) | SLTETTNQK | | **K**476**R** | | 51,17 | 5,31 | Subtype C |
|  | RT (560)-IN (8) | LFLDGIDKA | | **I**5**M** | | 0,26 | 13,43 | Not reported |
|  | IN 45 | Not within epitope | | **F**45**P** | | 19,56 | 0,12 |  |
|  | IN (241-250) | LLWKGEGAVV | | **V**249**M** | | 0,58 | 13,59 | Subtype B |
| 639 | PR (91-99) | TQIGCTLNF | | **I**93**L** | | 1,15 | 15,26 | Subtype B and C |
|  | RT (375-383) | IAMESIVIW | | **V**381**M** | | 97,49 | 16,22 | Subtype B and C |
|  | RT (550-559) | KLVSQGIRKV | | **K**550**R** | | 0,3 | 15,15 | Subtype A, B, C and D |
|  | IN (22-31) | MASDFNLPPIV | | **I**50**L** | | 2,95 | 19,08 | Not reported |
|  | IN (68-76) | LEGKIILVA | | **L**74**I** | | 1,63 | 14,39 | Subtype B |
| 641 | PR (11-20) | VTIKIGGQLK | | **G**17**D** | | 0,06 | 14,77 | Subtype B and C |
|  | PR (99)-RT (9) | FPISPIETVP | | **E**6**R** | | 0,07 | 12,53 | Subtype B |
|  | RT (73-82) | KLVDFRELNK | | **K**73**E** | | 0,01 | 15,72 | Subtype A, B and C |
|  | RT (94-102) | IPHPAGLKK | | **K**102**R** | | 0,25 | 13,37 | Subtype B |
|  | RT (181-189) | YQYMDDLYV | | **V**189**I** | | 13,84 | 22,48 | Subtype A, B, C and D |
|  | RT (233-241) | ELHPDRWTV | | **R**238**K** | | 14,34 | 0,11 | Subtype B and C |
|  | RT (272-280) | PGIKVRQLC | | **Q**278**H** | | 19,93 | 1,87 | Subtype B |
|  | RT (340-352) | QIYQEPFKNLKTG | | **I**341**V** | | 5,86 | 0,12 | Subtype B |
|  | RT (356-366) | KMRTAHTNDVK | | **M**357**R** | | 0,02 | 18,89 | Subtype B |
|  | RT (407-416) | QATWIPEWEF | | **E**415**D** | | 9,04 | 9,37 | Not reported |
|  | RT (416-425) | FVNTPPLVKL | | **V**417**I** | | 15,41 | 66,11 | Subtype C |
|  | RT (432-441) | EPIAGAETFY | | **P**433**L** | | 7,13 | 1,45 | Subtype C |
|  | RT (477-486) | TELQAIQLAL | | **Q**480**R** | | 10,46 | 24,32 | Subtype C |
|  | RT (526-534) | IKKEKIYLA | | **I**531**V** | | 0,05 | 18,05 | Subtype B |
|  | IN (22-31) | MASDFNLPPIV | | **L**27**S** | | 0,5 | 6,54 | Not reported |
|  | IN (123-131) | SAAVKAACWW | | **A**125**T** | | 6,81 | 1 | Subtype A, B, C and D |
|  | IN (241-250) | LLWKGEGAVV | | **V**249**M** | | 8,09 | 0,19 | Subtype B |
| 3387 | PR (4-14) | TLWQRPLVTIR | | **L**10**P** | | 7.76 | N/D | Subtype B |
|  | PR (43-52) | KPKMIGGIFI | | **G**49**R** | | 5.39 | N/D | Subtype B |
|  | PR (43-52) | KPKMIGGIFI | | **G**49**R** | | 5.15 | N/D | Subtype B |
|  | PR (53-61) | RNLMTQIGC | | **C**61**G** | | 8.43 | N/D | Subtype A and D |
|  | RT (5-12) | IETVPVKLK | | **K**15**R** | | 5.57 | N/D | Subtype B |
|  | RT (329-339) | IQKQGHGQWTY | | **H**334**Q** | | 15.79 | N/D | Subtype B |
|  | RT (509-518) | QPDKSESELV | | **E**416**K** | | 8.48 | N/D | Subtype B and C |
|  | RT (521-529) | IIEQLINKE | | **N**527**K** | | 17.75 | N/D | Subtype A |
|  | IN (22-31) | MASDFNLPPIV | | **A**23**V** | | 15.47 | N/D | Not reported |
| 843 | PR (34-42) | EEINLPGKW | | **P**39**V** | | 0,2 | 5,75 | Subtype C |
|  | PR (99)-RT (9) | FPISPIETVP | | **E**6**R** | | 15,95 | 22,81 | Subtype B |
|  | RT (5-12) | IETVPVKLK | | **K**10**R** | | 8,51 | 0,04 | Subtype B |
|  | RT (105-113) | SVTVLDVGD | | **V**106**K** | | 10,23 | 4,52 | Subtype B |
|  | RT (159-168) | IFQSSMTKIL | | **I**167**F** | | 7,01 | 0,14 | Subtype A, B and C |
|  | RT (159-168) | IFQSSMTKIL | | **I**167**F** | | 8,94 | 0,14 | Subtype A, B and C |
|  | RT (171-181) | FRAQNPEIVIY | | **V**179**D** | | 0,36 | 6,26 | Not reported |
|  | RT (304-312) | AENREILKE | | **R**307**K** | | 7043 | 0,04 | Subtype B and D |
|  | RT (340-352) | QIYQEPFKNLKTG | | **I**341**V** | | 0,17 | 8,98 | Subtype A and B |
|  | RT (379-388) | SIVIWGKTPK | | **K**388**R** | | 7,88 | 0,01 | Subtype B |
|  | RT (432-441) | EPIAGAETFY | | **I**434**M** | | 5,16 | 0,04 | Subtype C |
|  | RT (432-441) | EPIAGAETFY | | **F**440**Y** | | 3,46 | 5,61 | Subtype C |
|  | RT (449-457) | ETKLGKAGY | | **K**451**R** | | 12,06 | 5,97 | Subtype B |
|  | RT (477-486) | TELQAIQLAL | | **Q**480**R** | | 3,29 | 11,12 | Subtype C |
|  | IN (123-131) | SAAVKAACWW | | **A**124**T** | | 14,72 | 0,04 | Subtype A, B, C and D |
|  | IN (213-220) | LQKQITKI | | **T**218**L** | | 11,36 | 9,18 | Subtype B |
|  | IN (259-268) | VPRRKVKII | | **R**262**G** | | 7,34 | 0,04 | Subtype C |
| 1121 | PR (42-50) | WKPKMIGGI | | **K**43**R** | | 16,37 | 26,29 | Subtype B |
|  | PR (42-50) | WKPKMIGGI | | **K**45**R** | | 18,77 | 0,4 | Subtype B |
|  | PR (99)-RT (9) | FPISPIETVP | | **P**4**T** | | 0,28 | 7,43 | Subtype B |
|  | PR 31 | Not within epitope | | **S**31**N** | | 0,31 | 5,43 |  |
|  | RT (118-127) | VPLDEGFRKY | | **F**124**S** | | 8,64 | 0,35 | Subtype C |
|  | RT (192-202) | DLEIGQHRTKI | | **I**195**P** | | 0,26 | 6,17 | Subtype B |
|  | RT (203-212) | EELREHLLKW | | **E**207**R** | | 816 | 6,08 | Subtype C |
|  | RT (233-241) | ELHPDRWTV | | **R**238**K** | | 23,46 | 15,02 | Subtype B and C |
|  | RT (309-317) | ILKEPVHGV | | **P**313**S** | | 0,09 | 7,68 | Subtype B |
|  | RT (317-327) | VYYDPSKDLIA | | **S**322**T** | | 10,25 | 3,09 | Subtype C |
|  | RT (356-366) | KMRTAHTNDVK | | **R**358**K** | | 22,77 | 18,37 | Subtype B |
|  | RT (379-388) | SIVIWGKTPK | | **T**386**G** | | 14,22 | 66,38 | Subtype B |
|  | RT (379-388) | SIVIWGKTPK | | **T**386**G** | | 12,95 | 0,45 | Subtype B |
|  | RT (379-388) | SIVIWGKTPK | | **T**386**G** | | 8,85 | 3,43 | Subtype B |
|  | RT (397-406) | TWETWWTEYW | | **E**399**G** | | 1,21 | 6,1 | Subtype B |
|  | RT (449-457) | ETKLGKAGY | | **K**450**R** | | 0,81 | 14,18 | Subtype B |
|  | RT (461-469) | KGRQKIVTL | | **K**461**R** | | 0,3 | 8,65 | Subtype B and D |
|  | RT (461-469) | KGRQKIVTL | | **K**461**R** | | 2,58 | 8,1 | Subtype B and D |
|  | RT (461-469) | KGRQKIVTL | | **T**468**A** | | 2,31 | 10,31 | Subtype B and D |
|  | RT (550-559) | KLVSQGIRKV | | **Q**554**N** | | 15,48 | 14,4 | Subtype B and C |
|  | IN (22-31) | MASDFNLPPIV | | **D**25**E** | | 13,43 | 0,2 | Not reported |
|  | IN (78-86) | HVASGYIEA | | **S**81**T** | | 9,44 | 0,89 | Subtype B |
|  | IN (101-111) | ILKLAGRWPVK | | **K**111**R** | | 10,75 | 2,62 | Subtype C |
|  | IN (114-123) | HTDNGSNFTS | | **S**119**I** | | 1 | 5,51 | Subtype B |
| 6509 | RT (203-212) | EELREHLLKW | | **E**203**D** | | 7.32 | N/D | Subtype C |
|  | RT (203-212) | EELREHLLKW | | **E**204**K** | | 6.62 | N/D | Subtype C |
|  | RT (329-339) | IQKQGHGQWTY | | **I**329**V** | | 17.78 | N/D | Subtype B |
|  | RT (432-441) | EPIAGAETFY | | **A**435**G** | | 14.37 | N/D | Subtype C |
|  | RT (461-469) | KGRQKIVTL | | **G**462**E** | | 12.88 | N/D | Subtype B |
| 1475 | PR (42-50) | WKPKMIGGI | | **K**43**R** | | 0,13 | 7,7 | Subtype B |
|  | PR (75-84) | VLVGPTPVNI | | **V**82**G** | | 5,59 | 2,16 | Subtype A, B and C |
|  | PR (80-90) | TPVNIIGRNML | | **L**90**M** | | 8,61 | 0,3 | Subtype C |
|  | PR (91-99) | TQIGCTLNF | | **C**95**G** | | 0,02 | 8,39 | Subtype B and C |
|  | PR (99)-RT (9) | FPISPIETVP | | **P**4**T** | | 0,04 | 14,83 | Subtype B |
|  | PR (99)-RT (9) | FPISPIETVP | | **V**8**I** | | 11,93 | 0,45 | Subtype B |
|  | RT (113-120) | DAYFSVPL | | **V**118**S** | | 6,12 | 0,61 | Subtype C |
|  | RT (1336-144) | NNETPGIRY | | **I**142**L** | | 6,3 | 0,79 | Subtype C |
|  | RT (171-181) | FRAQNPEIVIY | | **N**175**H** | | 0,02 | 7,58 | Not reported |
|  | RT (171-181) | FRAQNPEIVIY | | **E**177**K** | | 0,07 | 8,47 | Not reported |
|  | RT (317-327) | VYYDPSKDLIA | | **D**320**S** | | 10 | 0,58 | Subtype C |
|  | RT (340-352) | QIYQEPFKNLKTG | | **I**341**V** | | 8,19 | 0,45 | Subtype B |
|  | RT (356-366) | KMRTAHTNDVK | | **T**359**E** | | 5,64 | 9,84 | Subtype B |
|  | RT (356-366) | KMRTAHTNDVK | | **T**359**E** | | 0 | 9,67 | Subtype B |
|  | RT (461-469) | KGRQKIVTL | | **T**468**A** | | 16,27 | 25,41 | Subtype B |
|  | RT (468-476) | SLTETTNQK | | **K**476**R** | | 35,48 | 12,12 | Subtype C |
|  | IN (9-19) | QEEHEKYHSNW | | **E**13**D** | | 14,61 | 4,85 | Subtype A, B and C |
|  | IN (22-31) | MASDFNLPPIV | | **D**25**E** | | 15,93 | 26,43 | Not reported |
|  | IN (101-111) | ILKLAGRWPVK | | **I**101**A** | | 8,74 | 0,28 | Subtype C and D |
|  | IN (135-146) | IQQEFGIPYNPQ | | **Y**143**C** | | 0,01 | 16,46 | Subtype C |
|  | IN (213-220) | LQKQITKI | | **Q**216**K** | | 10,03 | 97,95 | Subtype B |
| 6671 | RT (27-36) | TEEKIKALVE | | **K**32**T** | | 9.50 | N/D | Subtype A and B |
|  | RT (221-229) | HQKEPPFLW | | **E**224**G** | | 13.97 | N/D | Subtype B |
|  | RT (268-277) | SQIYPGIKVR | | **P**272**S** | | 13.54 | N/D | Subtype B and C |
|  | IN (23) | Not within epitope | | **L**23**M** | | 13.48 | N/D |  |
| 3253 | PR (11-20) | VTIKIGGQLK | | **G**17**D** | | 6,82 | 0,19 | Subtype C |
|  | PR (30-38) | DTVLEEMNL | | **E**35**D** | | 7,01 | 0,1 | Subtype B |
|  | PR (42-50) | WKPKMIGGI | | **K**43**R** | | 0,28 | 8,23 | Subtype B |
|  | PR (56-66) | VRQYDQIPIEI | | **V**56**F** | | 11,17 | 0,09 | Subtype B |
|  | PR (68-76) | GKKAIGTVL | | **I**72**V** | | 0,04 | 11,67 | Subtype B and C |
|  | PR (80-90) | TPVNIIGRNML | | **M**89**L** | | 9,01 | 0,22 | Subtype C |
|  | PR (91-99) | TQIGCTLNF | | **I**93**L** | | 5,66 | 55,38 | Subtype B and C |
|  | PR (99)-RT (9) | FPISPIETVP | | **E**6**R** | | 8,98 | 0,34 | Subtype B |
|  | RT (57-66) | NTPVFAIKKK | | **A**62**T** | | 13,21 | 0,89 | Subtype B and C |
|  | RT (113-120) | DAYFSVPL | | **Y**115**C** | | 0,01 | 6,98 | Subtype C |
|  | RT (113-120) | DAYFSVPL | | **V**118**S** | | 7,89 | 0,3 | Subtype C |
|  | RT (149-159) | LPQGWKGSPAI | | **A**158**S** | | 8,88 | 0,24 | Subtype C |
|  | RT (171-181) | FRAQNPEIVIY | | **N**175**H** | | 5,08 | 39,47 | Not reported |
|  | RT (203-212) | EELREHLLKW | | **R**206**K** | | 10,62 | 1,2 | Subtype C |
|  | RT (272-280) | PGIKVRQLC | | **Q**278**H** | | 5,84 | 46,85 | Subtype A, B and D |
|  | RT (304-312) | AENREILKE | | **R**307**K** | | 5,39 | 0,57 | Subtype B |
|  | RT (317-327) | VYYDPSKDLIA | | **I**326**V** | | 7,38 | 0,51 | Subtype C |
|  | RT (356-366) | KMRTAHTNDVK | | **T**359**E** | | 6,23 | 0,86 | Subtype B |
|  | RT (416-425) | FVNTPPLVKL | | **V**417**I** | | 15,1 | 0,21 | Subtype B and C |
|  | RT (449-457) | ETKLGKAGY | | **K**454**R** | | 5,4 | 0,92 | Subtype B |
|  | RT 503 | Not within epitope | | **H**503**Q** | | 6,57 | 0,4 |  |
|  | IN 23 | Not within epitope | | **L**23**I** | | 10,09 | 0,28 |  |
|  | IN (33-43) | AKEIVASCDKC | | **C**43**R** | | 0,02 | 7,88 | Not reported |
|  | IN (68-76) | LEGKIILVA | | **L**74**I** | | 14,89 | 0,15 | Subtype B |
|  | IN 252 | Not within epitope | | **S**252**V** | | 0,32 | 12,04 |  |
|  | IN (259-268) | VPRRKVKII | | **R**262**G** | | 10,49 | 0,2 | Subtype B and C |
|  | IN (278-288) | DDCVAGRQDED | | **D**288**N** | | 71,43 | 18,49 | Not reported |
| 3474 | PR (4-14) | TLWQRPLVTIR | | **I**13**V** | | 24,95 | 8,82 | Subtype B |
|  | RT (14-23) | PGMDGPKVKQ | | **V**21**I** | | 0,97 | 8,51 | Subtype B and C |
|  | RT 32 | Not within epitope | | **R**32**K** | | 71,35 | 18,98 |  |
|  | RT 33 | Not within epitope | | **D**33**G** | | 0,1 | 7,46 |  |
|  | RT (171-181) | FRAQNPEIVIY | | **Q**174**H** | | 0,63 | 15,79 | Not reported |
|  | RT (203-212) | EELREHLLKW | | **E**207**R** | | 0,6 | 8,38 | Subtype C |
|  | RT (240-248) | TVQPIVLPE | | **P**243**A** | | 0,84 | 17,13 | Subtype B |
|  | RT (340-352) | QIYQEPFKNLKTG | | **P**345**S** | | 0,13 | 6,74 | Subtype B |
|  | RT (379-388) | SIVIWGKTPK | | **T**386**G** | | 24,04 | 9,63 | Subtype B |
|  | RT (379-388) | SIVIWGKTPK | | **K**388**R** | | 23,47 | 9,1 | Subtype B |
|  | IN (101-111) | ILKLAGRWPVK | | **I**101**A** | | 0,28 | 16,32 | Subtype B and C |
| 7959 | PR (34-42) | EEINLPGKW | | **E**35**D** | | 6.09 | N/D | Subtype C and D |
|  | RT (203-212) | EELREHLLKW | | **E**207**D** | | 15.54 | N/D | Subtype C |
|  | RT (416-425) | FVNTPPLVKL | | **V**417**D** | | 15.39 | N/D | Subtype C |
|  | RT (432-441) | EPIAGAETFY | | **A**437**V** | | 12.75 | N/D | Subtype B and C |
|  | RT (461-469) | KGRQKIVTL | | **L**469**A** | | 17.89 | N/D | Subtype B |
|  | IN (267-275) | TLNETTNQK | | **N**273**K** | | 17.07 | N/D | Subtype A, B and D |
|  | IN (276) | Not within epitope | | **I**59**T** | | 14.99 | N/D |  |
|  | IN (68-76) | LEGKIILVA | | **I**73**V** | | 14.20 | N/D | Subtype C |
| 3910 | PR (11-20) | VTIKIGGQLK | | **G**17**D** | | 0,95 | 16,85 | Subtype C |
|  | PR (68-76) | GKKAIGTVL | | **K**70**Q** | | 10,08 | 0,6 | Subtype C |
|  | PR (80-90) | TPVNIIGRNML | | **L**90**M** | | 0,05 | 16,61 | Subtype A, B and C |
|  | RT (73-82) | KLVDFRELNK | | **K**73**E** | | 9,26 | 26,34 | Subtype C |
|  | RT (128-135) | TAFTIPSIN | | **I**134**V** | | 9,83 | 0,19 | Subtype B |
|  | RT (149-159) | LPQGWKGSPAI | | **G**155**S** | | 0,03 | 13,01 | Subtype C and D |
|  | RT 162 | Not within epitope | | **K**162**E** | | 0,36 | 18,06 |  |
|  | RT (304-312) | AENREILKE | | **I**309**T** | | 0,02 | 5,8 | Subtype B |
|  | RT (317-327) | VYYDPSKDLIA | | **D**319**S** | | 0,02 | 9,26 | Subtype C |
|  | RT (340-352) | QIYQEPFKNLKTG | | **K**350**R** | | 0,03 | 6,78 | Subtype B |
|  | RT (432-441) | EPIAGAETFY | | **P**433**L** | | 0,03 | 14,42 | Subtype B and C |
|  | RT (432-441) | EPIAGAETFY | | **A**435**V** | | 0,03 | 14,68 | Subtype B and C |
|  | RT (461-469) | KGRQKIVTL | | **V**467**I** | | 99,9 | 17,3 | Subtype B |
|  | RT (477-481) | TELQAIQLAL | | **Q**480**R** | | 14,41 | 0,46 | Subtype C |
|  | IN (22-31) | MASDFNLPPIV | | **L**27**S** | | 7,79 | 0,36 | Not reported |
|  | IN 252 | Not within epitope | | **L**252**V** | | 9,3 | 0,38 |  |
|  | IN (259-268) | VPRRKVKII | | **R**262**G** | | 0,07 | 6,56 | Subtype C and D |
|  | IN (278-288) | DDCVAGRQDED | | **E**287**D** | | 22,38 | 5,34 | Not reported |
| 9854 | RT (461-469) | KGRQKIVTL | | **G**462**E** | | 9.65 | N/D | Subtype B |
| 9915 | RT (116-124) | FSVPLDESF | | **S**123**R** | | 9.24 | N/D | Subtype B and C |
| 3869 | PR (68-76) | GKKAIGTVL | | **K**70**Q** | | 12,76 | 0,38 | Subtype C |
|  | RT (73-82) | KLVDFRELNK | | **K**73**E** | | 0,01 | 7,09 | Subtype B and C |
|  | RT (136-144) | NNETPGIRY | | **N**137**S** | | 0,01 | 6,89 | Subtype C |
|  | RT (171-181) | FRAQNPEIVIY | | **I**180**T** | | 0,02 | 6,64 | Subtype B |
|  | RT (203-212) | EELREHLLKW | | **E**204**K** | | 5,45 | 0,3 | Subtype A, B and C |
|  | RT (407-416) | QATWIPEWEF | | **E**415**D** | | 10,47 | 0,25 | Subtype B |
|  | RT (432-441) | EPIAGAETFY | | **P**433**L** | | 9,1 | 0,17 | Subtype C |
|  | RT (449-457) | ETKLGKAGY | | **L**452**V** | | 12,39 | 99,27 | Subtype B |
|  | RT (509-518) | QPDKSESELV | | **S**513**G** | | 0,04 | 6,28 | Subtype C and D |
|  | RT (519-527) | NQIIEQLIKK | | **K**526**Q** | | 7,78 | 0,32 | Subtype B |
|  | RT (550-559) | KLVSQGIRKV | | **Q**554**N** | | 15,92 | 0,4 | Subtype B and C |
|  | IN (22-31) | MASDFNLPPIV | | **L**28**S** | | 5,34 | 0,08 | Not reported |
|  | IN (101-111) | ILKLAGRWPVK | | **L**104**A** | | 11,32 | 1,36 | Subtype C |
|  | IN (101-111) | ILKLAGRWPVK | | **L**104**A** | | 5,62 | 0,2 | Subtype C |
|  | IN (114-123) | HTDNGSNFTS | | **S**119**I** | | 10,18 | 0,36 | Subtype A and B |
|  | IN (164-172) | QVRDQAEHL | | **D**167**E** | | 11,17 | 98,88 | Subtype C |
|  | IN (259-268) | VPRRKVKII | | **R**262**G** | | 7,91 | 0,3 | Subtype C |
| 3912 | PR (11-20) | VTIKIGGQLK | | **G**17**D** | | 5,25 | 0,44 | Subtype C |
|  | RT (221-229) | HQKEPPFLW | | **E**224**K** | | 16,71 | 1,3 | Subtype B |
|  | RT (233-241) | ELHPDRWTV | | **D**237**N** | | 18,86 | 19,46 | Subtype C |
|  | RT (375-383) | IAMESIVIW | | **V**381**M** | | 0,8 | 14,14 | Subtype B and C |
|  | RT (397-406) | TWETWWTEYW | | **E**399**G** | | 9,08 | 3,95 | Subtype B |
|  | IN 276 | Not within epitope | | **K**276**V** | | 0,17 | 15,96 |  |
| 3920 | PR (11-20) | VTIKIGGQLK | | **L**19**M** | | 8,43 | 0,28 | Subtype C |
|  | PR (56-66) | VRQYDQIPIEI | | **V**56**F** | | 0,72 | 8,54 | Subtype A, B and D |
|  | PR (80-90) | TPVNIIGRNML | | **M**89**L** | | 0,24 | 8,31 | Subtype C |
|  | PR (80-90) | TPVNIIGRNML | | **L**90**M** | | 5,41 | 0,14 | Subtype C |
|  | PR (42-50) | EKEGKISKI | | **I**50**S** | | 0,28 | 7,95 | Subtype B |
|  | PR 61 | Not within epitope | | **R**61**D** | | 26,21 | 18,08 |  |
|  | PR 79 | Not within epitope | | **S**79**G** | | 3,41 | 9,96 |  |
|  | RT (105-113) | SVTVLDVGD | | **V**106**K** | | 2 | 9,79 | Subtype B |
|  | RT (171-181) | FRAQNPEIVIY | | **Q**174**H** | | 11,13 | 0,66 | Subtype B and D |
|  | RT (192-202) | DLEIGQHRTKI | | **T**200**V** | | 5,59 | 0,19 | Subtype B |
|  | RT (203-212) | EELREHLLKW | | **R**206**K** | | 0,9 | 8,71 | Subtype C |
|  | RT (246-254) | LPEKDSWTV | | **D**250**E** | | 13,49 | 16,41 | Subtype C |
|  | RT (356-366) | KMRTAHTNDVK | | **R**358**K** | | 5,96 | 0,66 | Subtype B |
|  | RT (379-388) | SIVIWGKTPK | | **K**388**R** | | 0,26 | 5,11 | Subtype A, B and D |
|  | RT 395 | Not within epitope | | **I**395**M** | | 1,04 | 5,75 |  |
|  | RT (397-406) | TWETWWTEYW | | **E**399**G** | | 1,27 | 5,58 | Subtype B |
|  | RT (449-457) | ETKLGKAGY | | **L**452**V** | | 6,94 | 0,13 | Subtype B |
|  | RT (461-469) | KGRQKIVTL | | **I**466**M** | | 1,47 | 5,51 | Subtype B |
|  | RT (526-534) | IKKEKIYLA | | **E**529**K** | | 0,71 | 7,8 | Subtype B and D |
|  | RT 539 | Not within epitope | | **H**539**Q** | | 0,52 | 8,39 |  |
|  | IN (22-31) | MASDFNLPPIV | | **L**27**S** | | 2,43 | 7,44 | Not reported |
|  | IN (33-43) | AKEIVASCDKC | | **E**35**K** | | 0,63 | 8,05 | Subtype B |
|  | IN (164-172) | QVRDQAEHL | | **V**165**M** | | 42,7 | 18,82 | Subtype B and C |
|  | IN (164-172) | QVRDQAEHL | | **R**166**L** | | 2,08 | 8,9 | Subtype B and C |
|  | IN (164-172) | QVRDQAEHL | | **D**166**E** | | 4,02 | 11,11 | Subtype B and C |
|  | IN (185-194) | FKRKGGIGGY | | **G**189**R** | | 0,65 | 7,99 | Subtype C |
|  | IN 247 | Not within epitope | | **K**274**L** | | 0,93 | 8,31 |  |
| 9950 | PR (4-14) | TLWQRPLVTIR | | **L**10**P** | | 5.07 | N/D | Subtype B |
|  | PR (64-72) | IEICGHKAIG | | **E**64**G** | | 5.30 | N/D | Subtype A and B |
|  | IN (37-45) | VASCDKCQL | | **V**37**A** | | 15.68 | N/D | Subtype C |
|  | IN (96-105) | ETAYYILKLA | | **I**101**M** | | 14.46 | N/D | Subtype C |
|  | IN (165-172) | VRDQAEHL | | **D**167**E** | | 14.61 | N/D | Subtype A, B and C |
|  | IN (178-186) | MAVFIHNEK | | **E**187**S** | | 14.48 | N/D | Subtype B and D |
| 3935 | PR (56-66) | VRQYDQIPIEI | | **D**60**A** | | 0,92 | 8,97 | Subtype B |
|  | **PR (99)-RT (9)** | FPISPIETVP | | **P**4**T** | | 40,49 | 17,58 | Subtype B |
|  | **PR (99)-RT (9)** | FPISPIETVP | | **E**6**R** | | 5,43 | 8,98 | Subtype B |
|  | RT (5-12) | IETVPVKLK | | **K**10**R** | | 9,23 | 7,5 | Subtype C |
|  | RT (105-113) | SVTVLDVGD | | **V**106**K** | | 7,38 | 0,22 | Subtype B and D |
|  | RT (171-181) | FRAQNPEIVIY | | **E**177**K** | | 0,02 | 5,24 | Subtype B |
|  | RT (171-181) | FRAQNPEIVIY | | **I**180**T** | | 5,2 | 11,43 | Subtype B |
|  | RT (192-202) | DLEIGQHRTKI | | **I**195**P** | | 0,03 | 7,2 | Subtype A and C |
|  | RT (203-212) | EELREHLLKW | | **E**203**K** | | 5,86 | 0,4 | Subtype C |
|  | RT (203-212) | EELREHLLKW | | **E**204**K** | | 7,47 | 2,95 | Subtype C |
|  | RT (203-212) | EELREHLLKW | | **R**206**K** | | 5,38 | 0,47 | Subtype C |
|  | RT (203-212) | EELREHLLKW | | **E**207**R** | | 7,06 | 5,01 | Subtype C |
|  | RT (233-241) | ELHPDRWTV | | **D**237**N** | | 0,04 | 7,1 | Subtype B and C |
|  | RT (309-317) | ILKEPVHGV | | **P**313**S** | | 6,46 | 0,35 | Subtype B |
|  | RT (317-327) | VYYDPSKDLIA | | **D**320**S** | | 0,03 | 6,24 | Subtype C |
|  | RT (375-383) | IAMESIVIW | | **M**377**I** | | 5,76 | 5,89 | Subtype B and C |
|  | RT (375-383) | IAMESIVIW | | **E**378**S** | | 6,69 | 0,46 | Subtype B and C |
|  | RT (379-388) | SIVIWGKTPK | | **K**388**R** | | 11,73 | 0,22 | Subtype B |
|  | RT (407-416) | QATWIPEWEF | | **E**415**D** | | 0,04 | 7,88 | Subtype A and D |
|  | RT (416-425) | FVNTPPLVKL | | **K**424**T** | | 5,75 | 0,59 | Subtype C |
|  | RT (449-457) | ETKLGKAGY | | **K**454**R** | | 0,02 | 12,99 | Subtype B |
|  | RT (461-469) | KGRQKIVTL | | **T**468**A** | | 3,71 | 9,44 | Subtype B |
|  | RT (526-534) | IKKEKIYLA | | **A**534**T** | | 0,06 | 5,23 | Subtype A and B |
|  | IN (22-31) | MASDFNLPPIV | | **L**27**S** | | 8,79 | 6,9 | Not reported |
|  | IN (78-86) | HVASGYIEA | | **H**78**E** | | 0,08 | 6,69 | Subtype B |
|  | IN (123-131) | SAAVKAACWW | | **C**129**R** | | 0,02 | 5,62 | Subtype A, B, C and D |
|  | IN (185-194) | FKRKGGIGGY | | **R**187**K** | | 0,25 | 7,11 | Subtype C |
|  | IN (206-213) | TDIQTKEL | | **D**207**E** | | 7,86 | 0,82 | Subtype B and D |
|  | IN (278-288) | DDCVAGRQDED | | **G**283**S** | | 6,04 | 6,84 | Not reported |
| 4351 | PR (291-301) | IVPLTEEAEL | | **I**291**T** | | 18,94 | 0,29 | Subtype B |
|  | IN (22-31) | MASDFNLPPIV | | **L**27**S** | | 0,05 | 5,09 | Not reported |
| 5054 | PR (68-76) | GKKAIGTVL | | **I**72**V** | | 0,98 | 13,02 | Subtype C |
|  | PR (80-90) | TPVNIIGRNML | | **M**89**L** | | 0,42 | 8,06 | Subtype C |
|  | RT (159-168) | IFQSSMTKIL | | **I**167**F** | | 0,59 | 7,96 | Subtype A, B and C |
|  | RT (171-181) | FRAQNPEIVIY | | **N**175**H** | | 0,97 | 5,4 | Subtype C |
|  | RT (329-339) | IQKQGQGQWTY | | **I**329**K** | | 0,18 | 6,12 | Subtype B |
|  | RT (356-366) | KMRTAHTNDVK | | **V**365**A** | | 0,08 | 14,59 | Subtype B and D |
|  | RT (407-416) | QATWIPEWEF | | **E**415**D** | | 0,58 | 8,78 | Subtype B |
|  | RT (432-441) | EPIAGAETFY | | **F**440**Y** | | 0,86 | 16,41 | Subtype C and D |
|  | RT (519-527) | NQIIEQLIKK | | **N**519**S** | | 0,27 | 19,95 | Subtype B |
|  | RT 530 | Not within epitope | | **A**530**V** | | 0,13 | 8,13 |  |
|  | IN (22-31) | MASDFNLPPIV | | **D**25**E** | | 0,43 | 14,69 | Not reported |
|  | IN 51 | Not within epitope | | **F**51**P** | | 0,18 | 7,77 |  |
|  | IN (101-111) | ILKLAGRWPVK | | **L**104**A** | | 1,38 | 5,45 | Subtype C |
|  | IN (259-268) | VPRRKVKII | | **V**65**A** | | 0,43 | 8,55 | Subtype C |
| 6380 | RT (340-352) | QIYQEPFKNLKTG | | **K**350**R** | | 0,1 | 19,69 | Subtype B |
|  | IN (206-213) | TDIQTKEL | | **I**208**T** | | 11,58 | 0,47 | Subtype B |
| 6565 | PR (30-38) | DTVLEEMNL | | **E**35**D** | | 12,73 | 32,18 | Subtype B and D |
|  | PR (56-66) | VRQYDQIPIEI | | **D**60**A** | | 16,31 | 25,79 | Subtype B |
|  | RT (73-82) | KLVDFRELNK | | **K**73**E** | | 9,37 | 37,49 | Subtype C |
|  | RT 84 | Not within epitope | | **L**84**K** | | 12,38 | 3,42 |  |
|  | RT (181-189) | YQYMDDLYV | | **V**189**I** | | 12,8 | 4,2 | Subtype A, B and C |
|  | RT (317-327) | VYYDPSKDLIA | | **P**321**S** | | 11,86 | 0,97 | Subtype C |
|  | RT (317-327) | VYYDPSKDLIA | | **D**324**E** | | 12,3 | 85,63 | Subtype C |
|  | RT (329-339) | IQKQGQGQWTY | | **Q**334**N** | | 12,99 | 3,45 | Subtype B |
|  | RT (367-375) | QLTEAVHKI | | **A**371**V** | | 12,74 | 4,2 | Subtype C and D |
|  | RT (407-416) | QATWIPEWEF | | **E**415**D** | | 12,7 | 29,6 | Subtype B |
|  | IN (22-31) | MASDFNLPPIV | | **I**30**E** | | 13,23 | 4,2 | Not reported |
| 6596 | PR (80-90) | TPVNIIGRNML | | **M**89**L** | | 5,71 | 19,2 | Subtype C |
|  | RT (192-202) | DLEIGQHRTKI | | **I**195**P** | | 0,23 | 8,48 | Subtype B |
|  | RT (203-212) | EELREHLLKW | | **E**205**R** | | 13,67 | 3,4 | Subtype C |
|  | RT (203-212) | EELREHLLKW | | **E**205**R** | | 8,81 | 0,02 | Subtype C |
|  | RT (233-241) | ELHPDRWTV | | **D**237**N** | | 13,38 | 60,45 | Subtype B and C |
|  | RT (438-448) | ETFYVDGAANR | | **A**445**G** | | 1,35 | 8,94 | Subtype C |
| 2678 | PR (99)-RT (9) | FPISPIETVP | | **P**4**T** | | 28,66 | 7,15 | Subtype B |
|  | RT (461-469) | KGRQKIVTL | | **T**468**A** | | 0,67 | 6,26 | Subtype B |
|  | RT (491-501) | SEVNIVTDSQY | | **V**496**M** | | 13,21 | 97,2 | Subtype C and D |
|  | IN (33-43) | AKEIVASCDKC | | **E**35**K** | | 95,2 | 18,17 | Subtype B |
| 8828 | RT (118-127) | VPLDEGFRKY | | **F**124**S** | | 0,03 | 5,25 | Subtype C |
|  | RT 240 | Not within epitope | | **R**240**K** | | 5,91 | 96,2 |  |
|  | RT (367-375) | QLTEAVHKI | | **H**373**R** | | 0,19 | 14,68 | Subtype C |
|  | RT (519-527) | NQIIEQLIKK | | **N**519**S** | | 5,64 | 2,46 | Subtype A, B and D |
|  | RT (519-527) | NQIIEQLIKK | | **K**526**Q** | | 99,45 | 11,22 | Subtype A, B and D |
| 9986 | PR (30-38) | DTVLEEMNL | | **E**35**D** | | 95,83 | 2,56 | Subtype B and D |
|  | PR (68-76) | GKKAIGTVL | | **K**70**Q** | | 5,16 | 85,2 | Subtype C |
|  | PR (91-99) | TQIGCTLNF | | **C**95**G** | | 5,51 | 0,06 | Subtype C |
|  | RT (149-159) | LPQGWKGSPAI | | **A**158**S** | | 19,97 | 22,9 | Subtype A, C and D |
|  | RT 243 | Not within epitope | | **S**243**E** | | 6,68 | 0,04 |  |
|  | RT (356-366) | KMRTAHTNDVK | | **R**358**K** | | 9,56 | 5,12 | Subtype A and B |
|  | RT (379-388) | SIVIWGKTPK | | **K**388**R** | | 2,32 | 5,77 | Subtype B |
|  | RT (449-457) | ETKLGKAGY | | **L**452**V** | | 6,31 | 5,15 | Subtype B and D |
|  | RT (526-534) | IKKEKIYLA | | **I**531**K** | | 96,49 | 9,69 | Subtype B |
|  | RT (526-534) | IKKEKIYLA | | **A**534**T** | | 0,78 | 12,02 | Subtype B |
|  | RT (550-559) | KLVSQGIRKV | | **Q**554**N** | | 10,02 | 5,91 | Subtype C and D |

*Proportions outside of the range of analysis (below 5% and above 20%) were included to show the occurrence of the minority variant at different time point; for instance some increased or decreased with time. N/D = not done (some participants had no follow-up samples); RT = reverse transcriptase; PR = protease; IN = integrase; pol = polymerase; CTL = cytotoxic T-lymphocytes.
