# Supplementary material for: Deep sequencing of the HIV-1 polymerase gene for characterisation of cytotoxic T-lymphocyte epitopes during early and chronic disease stages
Source: Virol J. 2022 Mar 28;19:56. doi: 10.1186/s12985-022-01772-8 (PMC8959563; doi:10.1186/s12985-022-01772-8)
Supplement: Supplementary file 4 — Additional file 4. Supplementary Figure 1: Neighbour-joining phylogenetic analysis of Illumina consensus and Sanger consensus sequences for baseline samples. Sanger and Illumina consensus of the same sample significantly clustered together. Majority of the sequences clustered with HIV-1 subtype C references. HIV group O was used for rooting the tree and a bootstrap value of 1000 was used for analysis. S = Sanger sequencing; D = Deep sequencing. [file 12985_2022_1772_MOESM4_ESM.pdf]

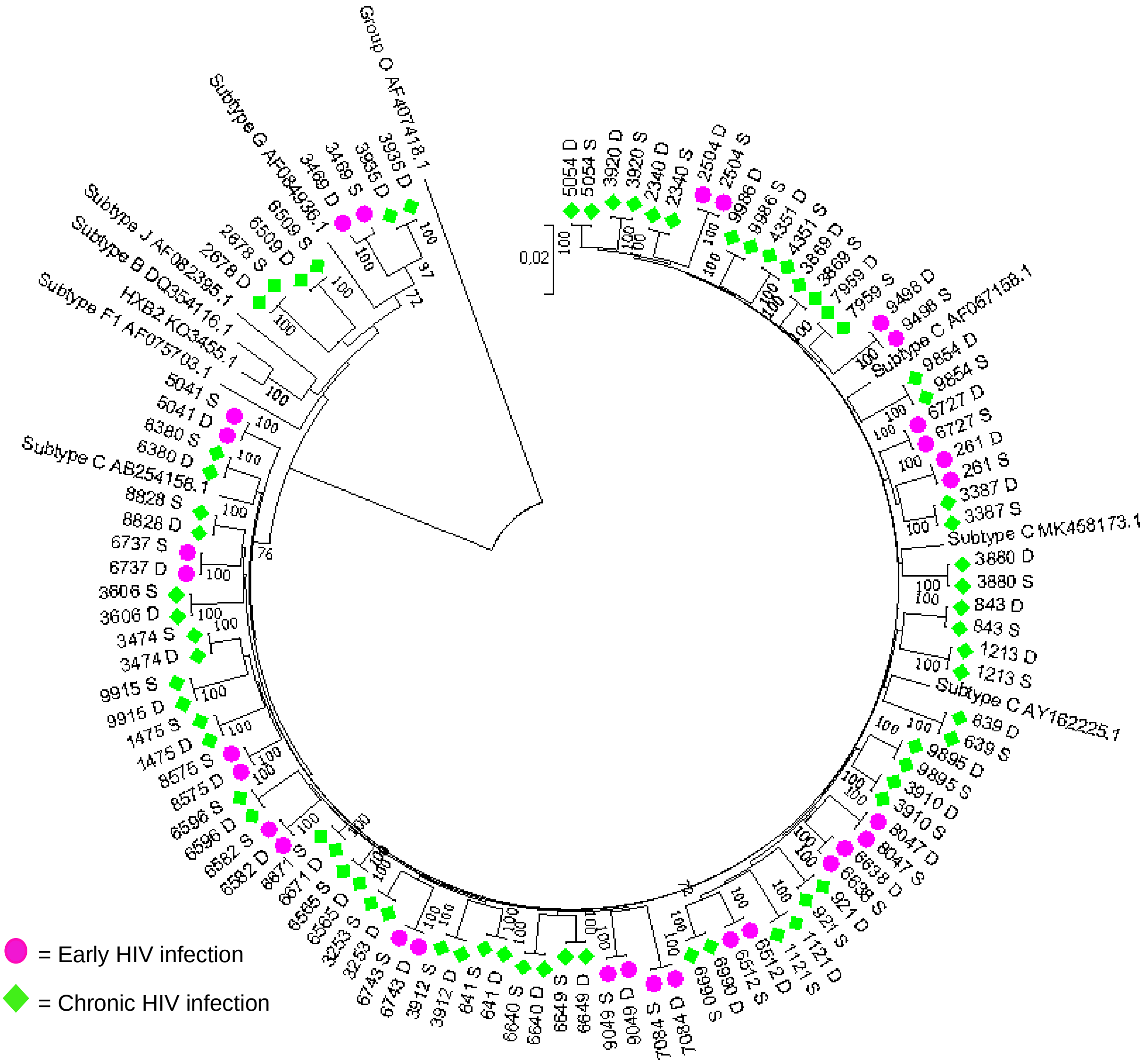

Supplementary Figure 1: Neighbour-joining phylogenetic analysis of Illumina consensus and Sanger consensus sequences for baseline samples. Sanger and Illumina consensus of the same sample significantly clustered together. Majority of the sequences clustered with HIV-1 subtype C references. HIV group O was used for rooting the tree and a bootstrap value of 1000 was used for analysis. S = Sanger sequencing; D = Deep sequencing.
